# Pan-cancer association of a centrosome amplification gene expression signature with genomic alterations and clinical outcome

**DOI:** 10.1371/journal.pcbi.1006832

**Published:** 2019-03-11

**Authors:** Bernardo P. de Almeida, André F. Vieira, Joana Paredes, Mónica Bettencourt-Dias, Nuno L. Barbosa-Morais

**Affiliations:** 1 Instituto de Medicina Molecular João Lobo Antunes, Faculdade de Medicina, Universidade de Lisboa, Lisboa, Portugal; 2 i3S - Instituto de Investigação e Inovação em Saúde, Universidade do Porto, Porto, Portugal; 3 IPATIMUP - Instituto de Patologia e Imunologia Molecular, Universidade do Porto, Porto, Portugal; 4 Instituto Gulbenkian de Ciência, Oeiras, Portugal; University of Maryland Baltimore County, UNITED STATES

## Abstract

Centrosome amplification (CA) is a common feature of human tumours and a promising target for cancer therapy. However, CA’s pan-cancer prevalence, molecular role in tumourigenesis and therapeutic value in the clinical setting are still largely unexplored. Here, we used a transcriptomic signature (CA20) to characterise the landscape of CA-associated gene expression in 9,721 tumours from The Cancer Genome Atlas (TCGA). CA20 is upregulated in cancer and associated with distinct clinical and molecular features of breast cancer, consistently with our experimental CA quantification in patient samples. Moreover, we show that CA20 upregulation is positively associated with genomic instability, alteration of specific chromosomal arms and C>T mutations, and we propose novel molecular players associated with CA in cancer. Finally, high CA20 is associated with poor prognosis and, by integrating drug sensitivity with drug perturbation profiles in cell lines, we identify candidate compounds for selectively targeting cancer cells exhibiting transcriptomic evidence for CA.

## Author summary

Centrosome amplification, i.e. an increased number of centrosomes—structures that exist inside cells, is a hallmark of cancer cells and therefore an Achilles' heel for the development of innovative therapies that specifically target tumour cells, sparing healthy ones. To exploit centrosome amplification’s clinical potential, it is crucial to understand its role in cancer development and to identify compounds for its selective targeting. These are challenging tasks due to the technical difficulty of profiling centrosome amplification in cells. In this study, we circumvent those challenges by computationally analysing the expression of 20 genes known to promote centrosome amplification across nearly 10,000 tumours of over 30 cancer types, thereby estimating their relative centrosome amplification levels. We found that those genes are indeed highly active in tumours and associated with prognosis in different cancer types. We also show that those genes’ expression is associated with instability in the structure of cancer cells’ chromosomes and identify candidate drugs for selectively targeting those cells. Our work therefore demonstrates the potential of computational analyses of large volumes of cancer molecular and clinical data to elucidate cellular and molecular mechanisms of tumour development and propose novel therapeutic options in oncology.

## Introduction

The centrosome, an organelle composed of two centrioles surrounded by a pericentriolar protein matrix, is the major microtubule-organising centre of animal cells, hence being pivotal for several fundamental cellular processes, including signalling, cell polarity, division and migration [1–4]. Each centrosome duplicates once per cell cycle to ensure bipolar spindle assembly and successful chromosome segregation [5,6]. Centrosomes are thus implicated in the maintenance of genome stability.

Centrosome amplification (CA)–the presence of more than two centrosomes—is a common feature in cancer [7]. Supernumerary centrosomes generate multipolar mitosis and consequent genome instability [6,8–10], they can accelerate and promote tumourigenesis *in vivo* [11–13] and promote cellular invasion and metastatic behaviour [14–17]. However, CA’s pan-cancer prevalence, molecular role in tumourigenesis and therapeutic value remain poorly understood, largely due to the technical challenges associated with profiling such small cellular structures in human cancer tissues. For instance, quantifying centrosome numbers and abnormalities is often hampered by the limited thickness of formalin-fixed and paraffin-embedded tissue sections, preventing the imaging of entire cells [18]. In addition, three-dimensional imaging and analysis are mandatory, but cumbersome and time consuming [19].

To at least partially circumvent those challenges, we propose the estimation of CA based on the expression levels of CA-associated genes. Recently, proof-of-principle gene-expression-based CA signatures have been developed [20–23], the most comprehensive one being CA20, based on the expression of *TUBG1*, which encodes the most abundant centrosomal protein, and 19 other genes whose overexpression has been experimentally shown to induce CA [23]. This signature was proposed to reflect CA levels in tumour samples and shown to have a prognostic value in two independent breast cancer cohorts [23].

In the present study, we used CA20 to estimate relative CA levels across 9,721 tumour and 725 matched-normal samples of 32 cancer types from The Cancer Genome Atlas (TCGA), thereby revealing the first pan-cancer landscape of CA-associated gene expression. We show the association of CA20 with distinct breast cancer clinical and molecular features. We also break down the independent associations of CA20 with different sorts of genomic instability across cancer types. Finally, we show that high CA20 is associated with poor clinical outcome in different cancer types, having identified candidate compounds for selectively targeting cancer cells exhibiting transcriptomic evidence for this hallmark of cancer.

## Results

### The pan-cancer landscape of centrosome amplification-associated gene expression

**Fig 1:**
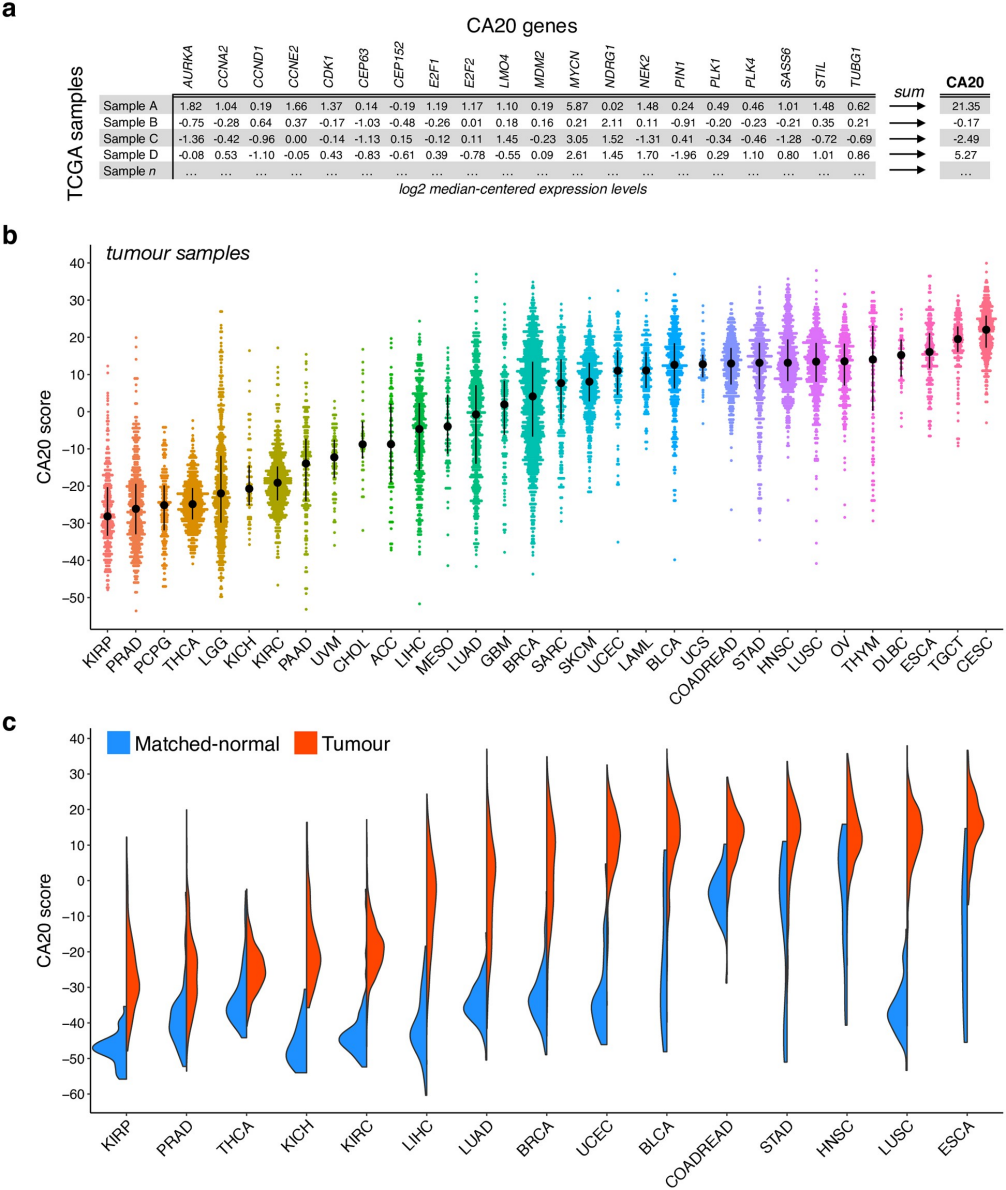
The pan-cancer landscape of centrosome amplification-associated gene expression. (**a**) For each sample, the CA20 score was calculated as the sum of the normalized (log2 median-centred) expression levels of the 20 signature genes. (**b**) CA20 score distribution across tumour samples of all TCGA cancer types. Cohorts are ordered by their median CA20 score. Black points and lines represent the median +/- upper/lower quartiles. (**c**) Tumour samples have higher CA20 levels in all 15 cancers with both tumour and matched-normal samples available (at least 10 samples per sample type; False Discovery Rate (FDR) < 0.0001, Wilcoxon rank-sum test). CA20 score distributions of tumour and normal samples are represented in red and blue, respectively. ACC: adrenocortical carcinoma; BLCA: bladder urothelial carcinoma; BRCA: breast invasive carcinoma; CESC: cervical squamous cell carcinoma and endocervical adenocarcinoma; CHOL: cholangiocarcinoma; COADREAD: colon and rectum adenocarcinoma; DLBC: lymphoid neoplasm diffuse large B-cell lymphoma; ESCA: oesophageal carcinoma; GBM: glioblastoma multiforme; HNSC: head and neck squamous cell carcinoma; KICH: kidney chromophobe; KIRC: kidney renal clear cell carcinoma; KIRP: kidney renal papillary cell carcinoma; LAML: acute myeloid leukemia; LGG: low-grade glioma; LIHC: liver hepatocellular carcinoma; LUAD: lung adenocarcinoma; LUSC: lung squamous cell carcinoma; MESO: mesothelioma; OV: ovarian serous cystadenocarcinoma; PAAD: pancreatic adenocarcinoma; PCPG: pheochromocytoma and paraganglioma; PRAD: prostate adenocarcinoma; SARC: sarcoma; SKCM: skin cutaneous melanoma; STAD: stomach adenocarcinoma; TGCT: testicular germ cell tumours; THCA: thyroid carcinoma; THYM: thymoma; UCEC: uterine corpus endometrial carcinoma; UCS: uterine carcinosarcoma; UVM: uveal melanoma.

To estimate relative CA levels in human samples, we used CA20, a score based on the expression of 20 genes experimentally associated with CA [23], as a surrogate. We quantified CA20 across the transcriptomes (profiled by RNA-seq) of 9,721 tumour and 725 matched-normal samples spanning 32 cancer types from TCGA (Fig 1a, S1 Table). CA20 correlates with the predicted proliferation rates of TCGA tumour samples [24] (Spearman’s correlation coefficient, r = 0.4, p-value < 2.2e-16; S1a Fig), as expected, given that some of the CA20 genes encode for proteins involved in cell proliferation. Cervical (CESC), testicular (TGCT) and oesophageal (ESCA) cancers show high CA20, contrasting with lower scores in kidney (KIRP, KICH and KIRC) and prostate (PRAD) cancers (Fig 1b). Some cancer types, such as low-grade glioma (LGG) and breast invasive carcinoma (BRCA), exhibit high variability of CA20, concordantly with previous observations that the proportion of cells with CA in breast tumours ranges from 1 to 100% [7,25] depending on the tumour subtype [26].

We also observed significant differences in CA20 between specific cancer types with the same tissue of origin. Although all kidney cancers have low CA20 scores, kidney renal papillary cell carcinoma (KIRP) shows a lower score than the other types (p-value < 0.0001, Wilcoxon rank-sum test; S1b Fig). Similarly, glioblastoma multiforme (GBM), skin cutaneous melanoma (SKCM) and lung squamous cell carcinoma (LUSC) show higher CA20 than low-grade glioma (LGG), uveal melanoma (UVM) and lung adenocarcinoma (LUAD), respectively (p-value < 0.0001 for all comparisons, Wilcoxon rank-sum test; S1b Fig). We note that squamous cell carcinomas have higher CA20 within cervical (CESC) and oesophageal (ESCA) cancers (p-value < 0.001 and < 0.01, respectively, Wilcoxon rank-sum test; S1c Fig), suggesting that the observed differences are indeed associated to the different cell types of origin and not only to differences between tissue of origin.

Since CA has been considered a hallmark of tumour cells [7], we tested the difference of CA20 between tumour and matched-normal samples. Indeed, tumour samples have higher CA20 levels in all 15 cancer types with both sample types available (at least 10 samples of each type; False Discovery Rate (FDR) < 0.0001, Wilcoxon rank-sum test; Fig 1c). In addition, using linear regression analyses with proliferation rate as an additional covariate, we found that CA20 is higher in tumour samples, either when considering all cohorts together (linear regression p-value < 0.0001, using cohort as an additional covariate; S2 Table) or per individual cohort (FDR < 0.0001 for all cohorts; S2 Table), independently of proliferation rate, discarding the suggestion of CA20 being its mere surrogate. These results emphasise CA as a hallmark of cancer.

### CA20 is associated with breast cancer clinical and molecular features

Breast cancer is one of the best studied cancer types, with large cohorts of clinically annotated tumour samples available [27,28], and where the CA20 score was developed [23]. In addition, CA has been frequently correlated with aggressive features in breast cancer [6,25,26,29]. Given that we observed high variability of CA20 in TCGA breast tumour samples, we sought to investigate in more detail the relationship between CA20 and different breast cancer molecular features in that cohort.

**Fig 2:**
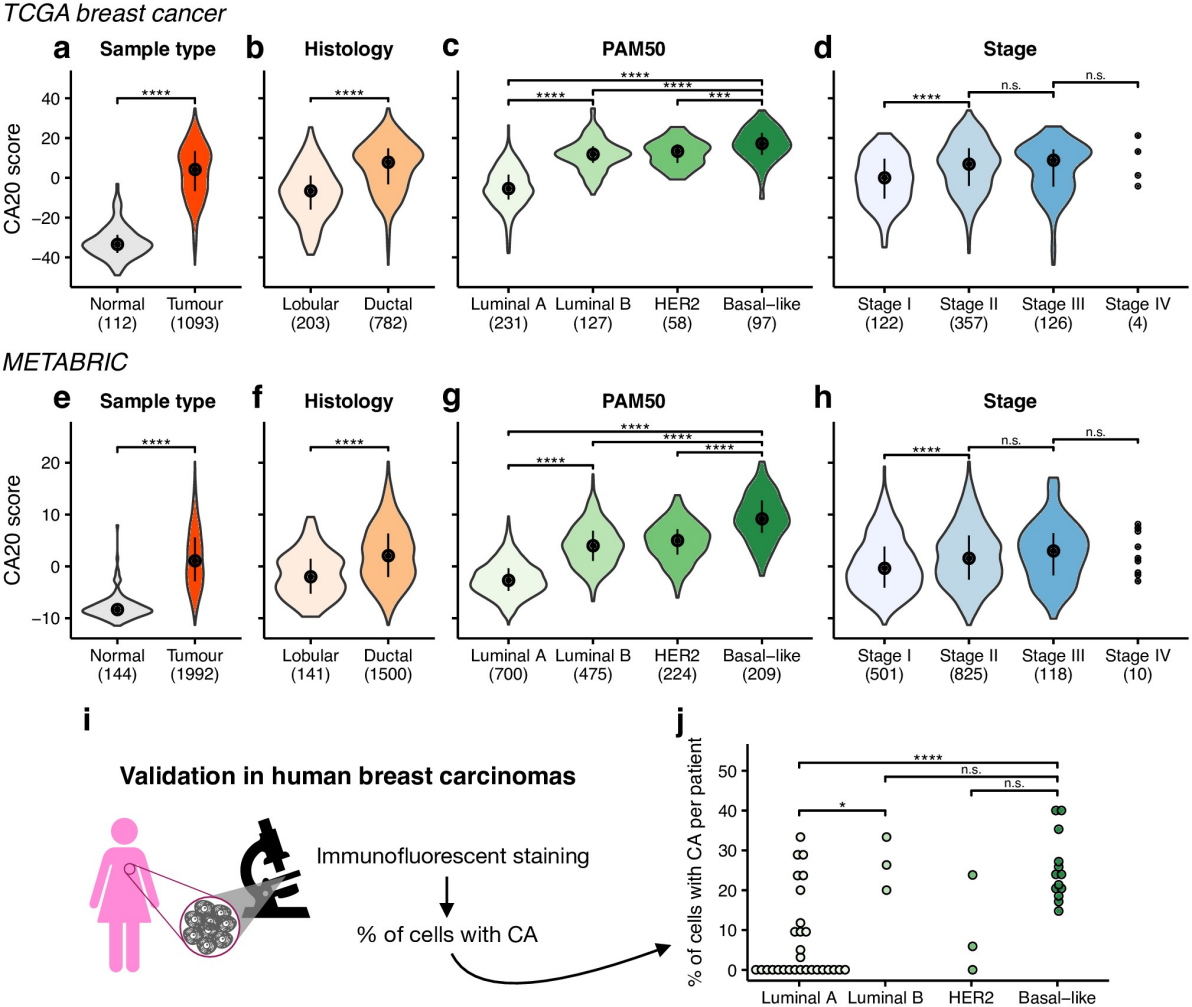
CA20 is associated with different breast cancer clinical and molecular features. **(a-h)** CA20 score distribution per (**a**,**e**) sample type, (**b**,**f**) histological and (**c**,**g**) PAM50 molecular subtype, and (**d**,**h**) tumour stage for (**a**-**d**) TCGA breast cancer and (**e**-**h**) METABRIC samples. Black points and lines represent the median +/- upper/lower quartiles. Number of samples used in each violin is shown within brackets. *** p-value < 0.001, **** p-value < 0.0001 and n.s. non-significant (Wilcoxon rank-sum test). **(i-j)** Luminal B and basal-like human breast carcinomas display higher levels of centrosome amplification (CA). **(i)** Illustration of the procedure to quantify CA in patient samples. **(j)** Percentage of cells displaying CA in breast tumours from the different PAM50 molecular subtypes (29 luminal A, 3 luminal B, 3 HER2 and 13 basal-like). Between 5 and 107 cells were analysed for each patient. * p-value < 0.05, *** p-value < 0.001 and n.s. non-significant (Wilcoxon rank-sum test).

CA20 is higher in tumours than in normal breast samples (p-value < 0.0001, Wilcoxon rank-sum test; Fig 2a) and we found higher levels of CA20 in invasive tumours from ductal histologic subtype (the most common, accounting for 90% of tumours) [30] when compared with lobular ones (p-value < 0.0001, Wilcoxon rank-sum test; Fig 2b). The difference between ductal and lobular subtypes is consistent in non-triple negative breast tumours (p-value < 0.0001, Wilcoxon rank-sum test; S2d Fig), as well as in samples from tumour stages II and III (p-value < 0.0001 and < 0.01, respectively, Wilcoxon rank-sum test; S2e Fig). We also tested the differences in CA20 between the different PAM50 molecular subtypes, derived based on a 50-gene classifier [31]. Basal-like breast tumours have the highest CA20 scores (p-value < 0.0001, p-value < 0.0001, and p-value < 0.001 for contrasts with luminal A, luminal B, and HER2-enriched, respectively, Wilcoxon rank-sum test; Fig 2c). This is in line with our recent work experimentally showing that basal-like breast cancers have indeed more CA than luminal ones [26]. We also observed a strong difference between luminal subtypes, with higher CA20 in luminal B samples (p-value < 0.0001, Wilcoxon rank-sum test; Fig 2c). Moreover, we tested the association between CA20 and tumour stage, having found a significant CA20 increase from stage I to stage II (p-value < 0.0001, Wilcoxon rank-sum test; Fig 2d), but no significant changes between subsequent stages (Fig 2d). All associations between CA20 and breast cancer histology, PAM50 molecular subtypes and tumour stage remain significant within both low and high proliferating tumours (samples divided by the median of estimated proliferation rates; S2a–S2c Fig).

All the aforementioned associations were validated in an independent cohort (Fig 2e–2h, S2f and S2g Fig and S3 Table), comprising 144 normal and 1,992 tumour breast samples from the Molecular Taxonomy of Breast Cancer International Consortium (METABRIC) [28]. We still tested the association between CA20 and the METABRIC integrative clusters, 10 molecular subgroups defined based on joint clustering of copy number and gene expression data [28]. CA20 varies across integrative clusters (p-value < 0.0001, Fligner-Killeen test) and is particularly enriched in cluster 10 (FDR < 0.0001, Wilcoxon rank-sum test, for comparisons with each of all the other clusters; S2h Fig), characterized by high proportion of basal-like tumours, high genomic instability, high rate of *TP53* mutations, chromosome arm 5q deletions and very poor prognosis in the short term [28].

We complementarily analysed the frequency of CA in human breast carcinomas from the different PAM50 molecular subtypes, comprising 29 luminal A, 3 luminal B, 3 HER2 and 13 basal-like tumours (Fig 2i and S4 Table). Concordantly with TCGA and METABRIC results, we observed a higher percentage of cells with supernumerary centrioles in luminal B (average of 27%) than in luminal A carcinomas (7%; p-value < 0.05, Wilcoxon rank-sum test; Fig 2j and S3 Fig). Moreover, basal-like (25%) display higher levels of CA than luminal A tumours (p-value < 0.0001, Wilcoxon rank-sum test). Despite the reduced number of luminal B samples, our patient data support CA20 as a good surrogate of CA levels and the suggestion that CA is more frequent in luminal B than in luminal A human breast carcinomas.

### CA20 is associated with genomic instability features in cancer

**Fig 3:**
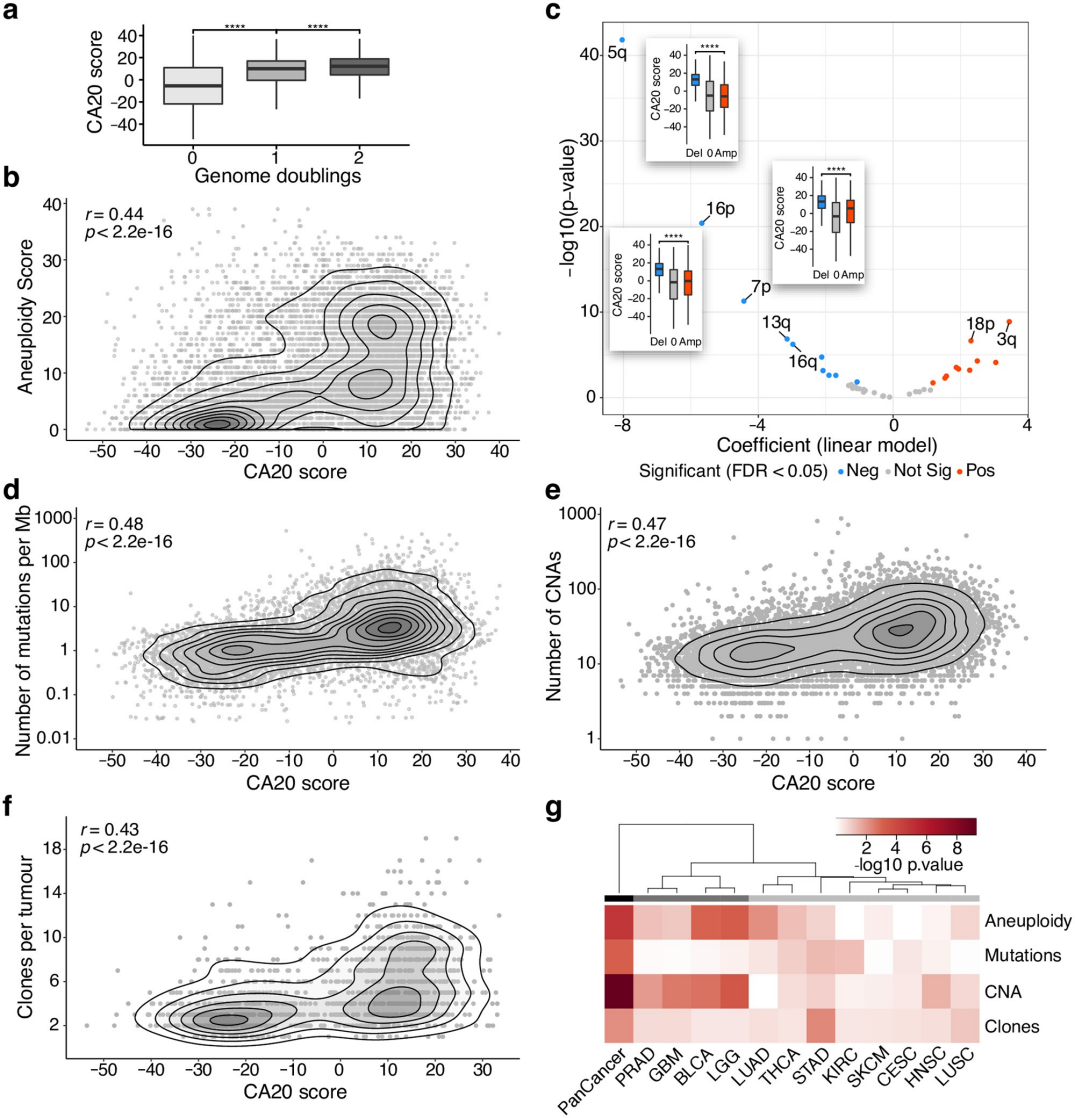
CA20 is associated with genomic instability features in cancer. (**a**) CA20 score is associated with genome doubling. Box plots of CA20 score per whole genome doubling status. **** p-value < 0.0001 (Wilcoxon rank-sum test). (**b**, **d**-**f**) CA20 is associated with different genomic instability features. Smooth scatter plots showing correlation between CA20 score and (**b**) aneuploidy score (measured as the total number of altered chromosome arms), (**d**) number of mutations per Mb, (**e**) number of CNAs and (**f**) clones per tumour across TCGA tumour samples (Spearman’s correlation coefficient, r = 0.44, 0.48, 0.47 and 0.43, respectively, p-value < 2.2e-16 for all). (**c**) Chromosome arm alterations associated with CA20 score. Volcano plot shows the results of linear regression analyses comparing CA20 score between samples with deletion or amplification of each chromosome arm. Arms whose deletions or amplifications are associated with higher CA20 (FDR < 0.05) are represented in blue and red, respectively. Chromosome arms with FDR < 1e-5 are highlighted and box plots of CA20 score per chromosome arm alteration are shown for 5q, 16p and 7p arms. **** p-value < 0.0001 (linear regression). (**g**) Hierarchical clustering of TCGA cancer types based on the independent association between the different genomic instability features and CA20 score. Unsupervised hierarchical clustering using Euclidean distances calculated based on multiple linear regression p-values of association with CA20 of aneuploidy score, number of mutations per Mb, number of CNAs and clones per tumour, per TCGA cohort and with all cohorts together (PanCancer). Heatmap colour scale according with -log10 of linear regression p-values. Main clusters are highlighted with different shades of grey. BLCA: bladder urothelial carcinoma; CESC: cervical squamous cell carcinoma and endocervical adenocarcinoma; GBM: glioblastoma multiforme; HNSC: head and neck squamous cell carcinoma; KIRC: kidney renal clear cell carcinoma; LGG: low-grade glioma; LUAD: lung adenocarcinoma; LUSC: lung squamous cell carcinoma; PRAD: prostate adenocarcinoma; SKCM: skin cutaneous melanoma; STAD: stomach adenocarcinoma; THCA: thyroid carcinoma.

CA and consequent multipolar mitoses have been associated with aneuploidy, genomic instability and tumourigenesis for more than a century [32,33]. Using the available quantitative characterization of aneuploidy in TCGA [34], we found that CA20 is higher in samples with genome doubling (p-value < 0.0001, Wilcoxon rank-sum test; Fig 3a) and positively correlated with their aneuploidy score (measured as the total number of altered—gained or lost—chromosome arms; Spearman’s correlation coefficient, r = 0.44, p-value < 2.2e-16; Fig 3b). Although CA20 is positively correlated with both chromosomal deletions and amplifications (Spearman’s correlation coefficient, r = 0.41 and 0.36, p-value < 2.2e-16, respectively; S4a and S4b Fig), it is more strongly associated with chromosomal deletions (p-value < 2.2e-16, t-test for z-transformed coefficients; see also S4c Fig). Given the known association between loss of p53 and CA [6,7,35] and the recent observation that p53 null cells tend to have an enrichment of chromosome losses over gains [36], we tested the hypothesis that the observed association between CA20 and chromosomal deletions could be linked to *TP53* mutations. However, the increase in the proportion of deletions per sample from low to high CA20 samples is consistent within both *TP53* wild-type and mutated samples (p-value < 0.0001 and < 0.05, respectively, Wilcoxon rank-sum test; S4d and S4e Fig), showing it is independent of *TP53* mutations (two-way ANOVA p-value for interaction = 0.6; S4d Fig).

Investigating the hypothesis that CA20-associated aneuploidy levels could vary between chromosomes, we identified 20 chromosome arms whose deletion (10 arms) or amplification (10 arms) was enriched in tumour samples with higher CA20 (linear regression, FDR < 0.05; Fig 3c and S2 and S5 Tables). The strongest associations were with loss of 5q, 16p and 7p. Interestingly, 5q deletion was previously associated with CA20-high basal-like breast tumours [27,37–40] and METABRIC integrative cluster 10 [28] (Fig 2c and 2g and S2h Fig). The association between CA20 and 5q deletion remains when removing the breast cancer cohort (linear regression p-value < 2.2e-16; S5 Fig and S2 Table). This observation raises the question if matched-normal samples of the analysed tumour samples have a CA20 signal predictive of those 5q, 16p and 7p deletions. We tested this hypothesis by comparing the CA20 levels between normal samples (with intact tested chromosomal arms) whose matched tumours lost 5q, 16p or 7p, with those with tumours with amplifications or no alterations in those chromosomal arms. We found that normal samples whose matched tumours lost 5q or 16p exhibit higher CA20 scores (p-value < 0.01 and < 0.05, respectively, Wilcoxon rank-sum test; S6 Fig), therefore suggesting that a CA20 increase may precede those chromosomal abnormalities.

In addition to tumour aneuploidy, CA20 is positively correlated with mutation burden, number of somatic Copy Number Alterations (CNA) and number of clones per tumour (Spearman’s correlation coefficient, r = 0.48, 0.47 and 0.43, respectively, p-value < 2.2e-16 for all; Fig 3d–3f). All these associations are independent of cell proliferation (linear regression p-values < 1e-8 for all; S2 Table and S7 Fig). We found that the correlation with mutation burden holds for different types of mutations (silent, missense, splice site and nonsense), as well as for mutations shown to be pathogenic (data from ClinVar https://www.ncbi.nlm.nih.gov/clinvar/) in all diseases and particularly in cancer (S8 Fig). Since these genomic instability features are likely correlated between each other, we applied multiple linear regression analyses across 1050 tumour samples (from 12 different cancer types; minimum of 30 and average of 88 samples per cohort) with information for those 4 covariates (S6 Table). We identified independent positive associations between CA20 and all genomic instability features, with stronger association for CNAs (linear regression p-values = 1.3e-5, 7.2e-4, 5.3e-10 and 6.4e-3 for aneuploidy, mutation burden, CNA and number for clones, respectively; Fig 3g and S2 Table). These associations remain significant when proliferation rate is used as an additional covariate in the regression (p-values = 2.3e-5, 7e-4, 2.4e-9 and 0.03 for aneuploidy, mutation burden, CNA and number for clones, respectively; S2 Table). We performed similar analyses per TCGA cohort and identified a group of cancer types where CA20 is mostly associated with CNA and aneuploidy (prostate adenocarcinoma, glioblastoma multiforme, bladder urothelial carcinoma, and brain low-grade glioma; Fig 3g and S9 Fig; S2 Table). Although CA has been globally associated with genomic instability, these results highlight CNA as the main associated feature and show that these associations differ between cancer types.

### CA20 is associated with cancer’s mutational spectrum

**Fig 4:**
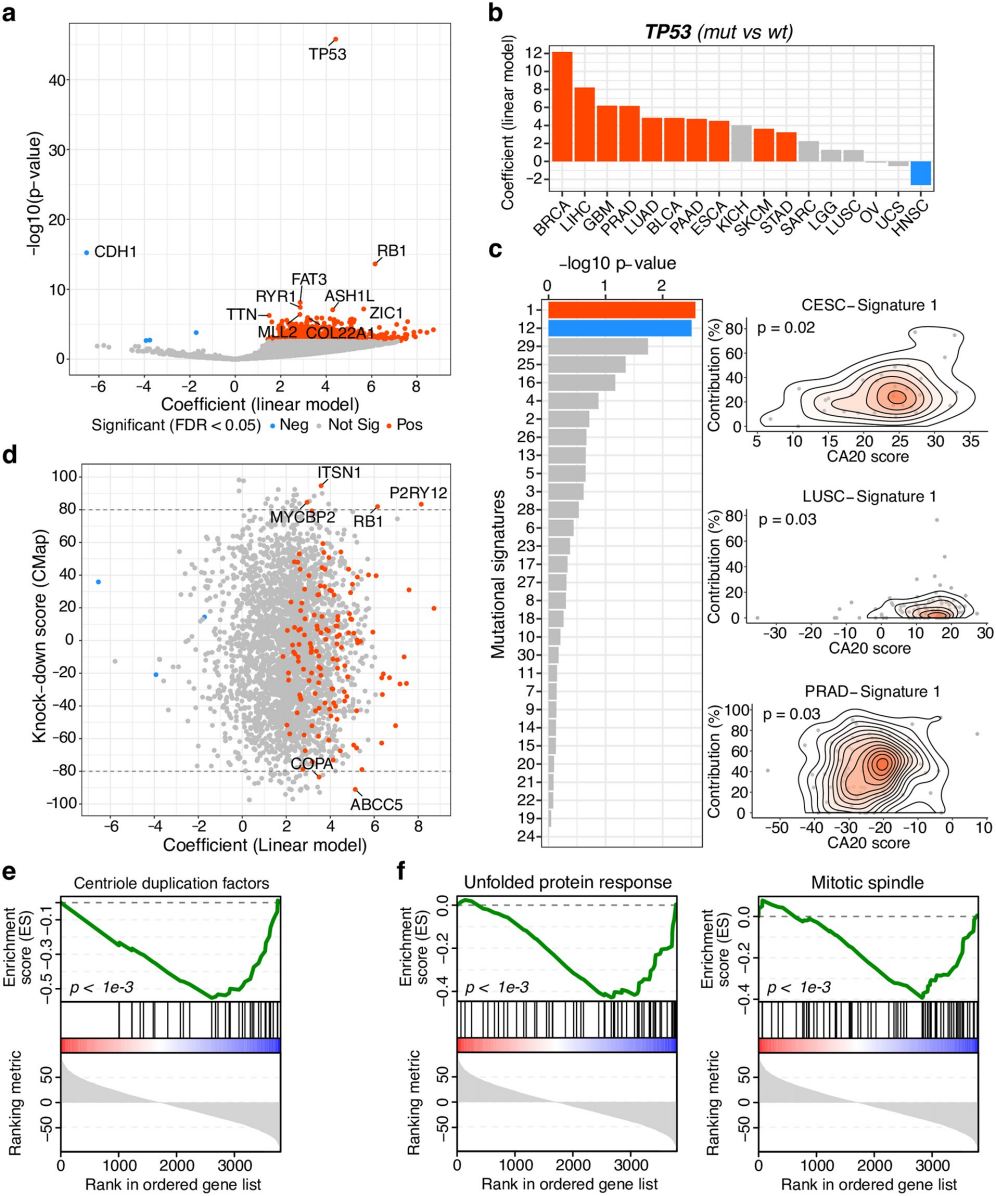
CA20 is associated with cancer’s mutational spectrum. (**a**) Somatic mutations pan-cancer-wide associated with the CA20 score. The volcano plot shows the results of linear regression analyses comparing the CA20 score between mutated and wild-type samples for 14,589 genes (at least 20 mutated samples). Genes whose mutations are associated with higher and lower CA20 (FDR < 0.05) are represented in red and blue, respectively. The top 10 genes are highlighted. (**b**) *TP53* mutations are associated with CA20 in different cancer types. Linear regression coefficients, representing CA20 score differences between *TP53* mutated and wild-type tumour samples, across TCGA cohorts with at least 20 mutated samples. Significant associations (FDR < 0.05) are coloured as in (a). (**c**) Mutational signatures pan-cancer-wide associated with CA20, independently of other types of genomic instability. Left: Significance of linear regression analyses (-log10 p-value) between CA20 and contribution of each mutational signatures including, as independent variables, the four genomic instability features: aneuploidy, mutation burden, CNA and number of clones per tumour. Positive and negative significant associations (FDR < 0.05) are coloured in red and blue, respectively. Right: Smooth scatter plots showing correlations between CA20 and the contribution of mutational signature 1 (linked with ageing) in 3 TCGA cohorts. Linear regression p-values are shown. (**d**) Causal effect of CA20-associated mutations on CA20 levels. Scatter plot of linear regression’s coefficient from (a) versus Connectivity Map (CMap)’s knock-down score, ranging from 100 (CA20 up-regulation) to -100 (CA20 down-regulation), for each gene in common. Genes are coloured as in (a) and the ones with both significant linear regression associations and absolute knock-down score higher than 80 are highlighted. (**e** and **f**) Gene Set Enrichment Analysis (GSEA) of genes ranked by their CMap’s knock-down score using (**e**) a manually curated list of centriole duplication factors and (**f**) MSigDB’s Hallmark Gene Sets (unfolded protein response and mitotic spindle were significantly associated, FDR < 0.05). GSEA p-values are shown. BLCA: bladder urothelial carcinoma; BRCA: breast invasive carcinoma; CESC: cervical squamous cell carcinoma and endocervical adenocarcinoma; ESCA: oesophageal carcinoma; GBM: glioblastoma multiforme; HNSC: head and neck squamous cell carcinoma; KICH: kidney chromophobe; LGG: low-grade glioma; LIHC: liver hepatocellular carcinoma; LUAD: lung adenocarcinoma; LUSC: lung squamous cell carcinoma; OV: ovarian serous cystadenocarcinoma; PAAD: pancreatic adenocarcinoma; PRAD: prostate adenocarcinoma; SARC: sarcoma; SKCM: skin cutaneous melanoma; STAD: stomach adenocarcinoma; UCS: uterine carcinosarcoma.

Point mutations are one of the most common types of mutational events that impact the stability of a cancer genome. We examined the pan-cancer association between CA20 and somatic mutations in 14,589 genes and found 752 whose mutations are associated with CA20 (FDR < 0.05; Fig 4a and S2 and S7 Tables). Most significant associations of mutated genes with the CA20 score are positive, consistently with its correlation with higher mutation burden (Fig 3d), and enriched in cancer driver genes (Gene Set Enrichment Analysis (GSEA) [41,42] p-value < 0.001, using a list of 299 cancer driver genes derived from TCGA’s PanCancer analysis [43]; S10a Fig). *TP53* shows the strongest association (linear regression p-value < 0.0001; Fig 4a), with positive correlations for the majority of cancer types surveyed (10 out of 17 cancer types with at least 20 mutated samples; FDR < 0.05; Fig 4b), therefore putatively extending the reported association between loss of p53 and CA [6,7,35] to 10 different cancer types. The second strongest positive association is with tumour suppressor pRb (*RB1*), whose acute loss has been found to induce CA [44]. Unexpectedly, the strongest negative association is with E-cadherin (encoded by *CDH1*), meaning *CDH1*-mutated samples have lower CA20 levels. Given its tumour suppressor role in cancer and the fact that its mutations mostly induce loss of function [45], this result suggests loss of E-cadherin is associated with lower CA in human tumours, which is contrary to what have been reported in epithelial cancer cells [46]. GSEA on genes whose mutations are associated with CA20 found that they are enriched in cancer-associated pathways and Wnt/β-catenin signalling (S10c–S10f Fig). As only a small fraction of somatic mutations represent driver events, we repeated the pan-cancer analysis of association between CA20 and somatic mutations using likely driver mutations from the Cancer Genome Interpreter (https://www.cancergenomeinterpreter.org/mutations) [45]. Within the tested 33 genes with at least 10 mutated samples, we found three (*TP53*, *PIK3CA* and *EGFR*) whose driver mutations are associated with CA20 (FDR < 0.05; S10b Fig and S2 and S8 Tables), *TP53* being again the strongest association. Overall, we show that CA20 is associated with both passenger and driver mutational spectra in cancer, with particular enrichment in cancer driver genes and Wnt/β-catenin signalling.

CA has still been proposed as a driver of genomic instability [11]. We thus wondered if the DNA mutation spectrum associated with CA was similar to specific signatures of somatic mutations caused by different mutational processes in cancer [47]. We therefore retrieved the contribution of the 30 published mutational signatures for each TCGA tumour sample from mSignatureDB [48] and uncovered three of them positively associated with CA20: signature 3, associated with BRCA1/2 mutations; signature 13, attributed to APOBEC activity; and signature 4, characteristic of smoking’s mutational pattern (FDR < 0.05; S11 Fig). As these signatures are likely confounded with genomic instability, we performed multiple linear regression on CA20 including, as independent variables, the mutational signature and the four aforementioned genomic instability features: aneuploidy, mutation burden, CNA and number of clones per tumour (S2 Table). Signature 1, linked with ageing and characterised by C>T substitutions (S12a Fig), and its “reverse” (T>C substitution bias) Signature 12, found mainly in liver cancer (S12b Fig), are respectively positively and negatively associated (FDR < 0.05) with CA20 (Fig 4c), independently of other types of genomic instability and even when proliferation rate is added as a variable (FDR = 0.051 for both signatures).

To evaluate the putative causality of CA20-associated mutations (Fig 4a), we interrogated the Connectivity Map (CMap) database of signatures [49] about the impact of each of the 3,799 gene knock-downs on the CA20 gene set in human cancer cell lines. The resultant connectivity scores (S9 Table), ranging from 100 (CA20 up-regulation) to -100 (CA20 down-regulation), were compared with the pan-cancer association between somatic mutations in the cognate genes and CA20 (Fig 4d). We thereby identified 6 genes with a putative causal effect on CA20 scores (|connectivity score| > 80; Fig 4d): *P2RY12*, *RB1*, *ITSN1* and *MYCBP2* are putative inhibitors of CA (their knock-down up-regulate CA20 genes), whereas *ABCC5* and *COPA* are putative promoters of CA (their knock-down down-regulate CA20 genes). Although acute loss of pRb (encoded by *RB1*) has been found to induce CA [44], confirming pRb as a CA inhibitor, to our knowledge none of the remaining genes identified herein has been previously associated with CA. They are therefore interesting candidates for future functional studies. Genes from a manually curated list of centriole duplication factors (93 genes, including only 10 from the CA20 signature; S10 Table) are enriched in negative CMap knock-down scores (GSEA p-value < 0.001; Fig 4e), suggesting they are indeed needed for cells to express CA-associated genes. Using the MSigDB’s Hallmark Gene Sets library [50], we identified unfolded protein response and mitotic spindle as significantly enriched in genes whose knock-down showed negative scores, i.e. CA20 down-regulation (GSEA FDR < 0.05; Fig 4f). This association suggests that mitotic spindle components activate CA-associated genes and/or that cells highly expressing CA-associated genes may be less likely to survive when their mitotic spindle is perturbed.

### CA20 is associated with prognosis, hypoxia and stromal infiltration in cancer

**Fig 5:**
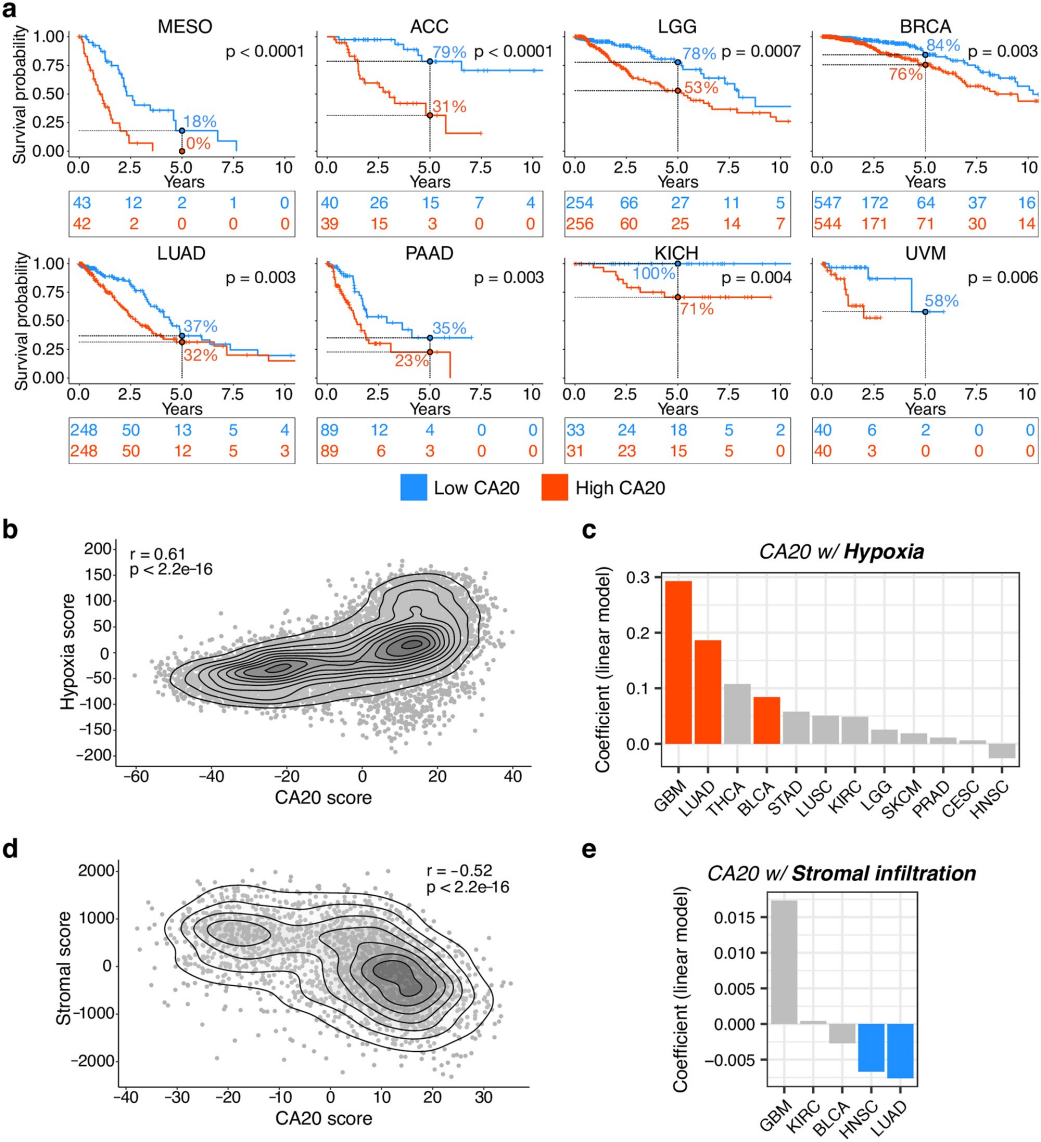
High CA20 is associated with poor patient prognosis, hypoxia and lower stromal infiltration in cancer. (**a**) Kaplan-Meier plots for patient stratification based on CA20 score (patients divided by CA20 median: lower CA20 in blue and higher CA20 in red) in 8 different cancer types. Numbers at risk every 2.5 years (tables) and 5-year survival rates (points and dashed lines) are shown. P-values for log-rank tests for differences in survival are shown. (**b**) CA20 upregulation is associated with hypoxia. Smooth scatter plot showing correlation between the hypoxia and the CA20 scores across TCGA tumour samples (Spearman’s correlation coefficient, r = 0.61, p-value < 2.2e-16). (**c**) CA20 upregulation is associated with hypoxia in different cancer types. Linear regression coefficients, representing the CA20 score dependence on hypoxia score, independently of genomic instability, across the TCGA cohorts with information for all covariates. Significant associations (FDR < 0.05) are coloured. (**d**) CA20 is associated with lower stromal cell infiltration. Smooth scatter plot showing correlation between the CA20 and the stromal scores across TCGA tumour samples (Spearman’s correlation coefficient, r = -0.52, p-value < 2.2e-16). (**e**) CA20 is associated with lower stromal cell infiltration in head and neck squamous cell carcinoma and lung adenocarcinoma. Linear regression coefficients, representing the CA20 score dependence on stromal score, independently of genomic instability, across the TCGA cohorts with information for all covariates. Significant associations (FDR < 0.05) are coloured. ACC: adrenocortical carcinoma; BLCA: bladder urothelial carcinoma; BRCA: breast invasive carcinoma; CESC: cervical squamous cell carcinoma and endocervical adenocarcinoma; GBM: glioblastoma multiforme; HNSC: head and neck squamous cell carcinoma; KICH: kidney chromophobe; KIRC: kidney renal clear cell carcinoma; LGG: low-grade glioma; LUAD: lung adenocarcinoma; LUSC: lung squamous cell carcinoma; MESO: mesothelioma; PAAD: pancreatic adenocarcinoma; PRAD: prostate adenocarcinoma; SKCM: skin cutaneous melanoma; STAD: stomach adenocarcinoma; THCA: thyroid carcinoma; UVM: uveal melanoma.

CA has been associated with poor patient prognosis in a variety of cancer types [7]. We therefore tested CA20’s association with overall patient’s survival across 31 TCGA cancer types with more than 40 samples each, finding high CA20 significantly associated with worse prognosis in 8 different cancer types (FDR < 0.05, log-rank test; Fig 5a and S11 Table). This result supports the potential of CA20 for prognostic-based patient stratification.

Hypoxia is a potent microenvironmental factor promoting genetic instability and malignant progression [51–53]. Given that hypoxia has been shown to enhance centrosome aberrations in breast cancer [54,55], we investigated whether CA20 is associated with the relative hypoxia levels in TCGA tumour samples, given by a previously established surrogate metagene expression signature [56]. We found a positive correlation between CA20 and the hypoxia score (Spearman’s correlation coefficient, r = 0.61, p-value < 2.2e-16; Fig 5b) that is independent of genomic instability (linear regression p-value = 7.8e-9; S2 Table). We further confirmed that this association is independent of estimated proliferation rates (linear regression p-value = 5.6e-7 when proliferation rate is added as a covariate to the regression; S13a Fig and S2 Table). We also performed this linear regression analysis for each of the 12 TCGA cohorts with information for all covariates and identified three cancer types (glioblastoma multiforme, lung adenocarcinoma and bladder urothelial carcinoma) where hypoxia is positively associated (FDR < 0.05) with CA20 (Fig 5c; S2 Table).

Although a tumour is also composed by stromal and immune cells [57], the association between CA and tumour cellular composition has not been addressed yet. CA20 is associated with lower stromal (Spearman’s correlation coefficient, r = -0.52, p-value < 2.2e-16; Fig 5d) and immune (Spearman’s correlation coefficient, r = -0.34, p-value < 2.2e-16; S13c Fig) cell infiltration in TCGA. However, pan-cancer linear regression analyses revealed that only the negative association with stromal infiltration is independent of genomic instability (linear regression p-value = 2.7e-6 and 0.24, for stromal and immune, respectively; S2 Table). The same was observed when including proliferation rate as an additional covariate (linear regression p-value = 1.2e-4 and 0.21, respectively; S13b and S13d Fig and S2 Table). We have also performed similar analyses for each of the 5 TCGA cohorts with information for all covariates and found that CA20 is significantly associated (FDR < 0.05) with lower stromal infiltration in head and neck and lung cancers (Fig 5e), with lower immune infiltration in glioblastoma, and with higher immune infiltration in head and neck cancer (S13e Fig), all independently of genomic instability (S2 Table).

### Identification of compounds that selectively kill cancer cells with high CA20

**Fig 6:**
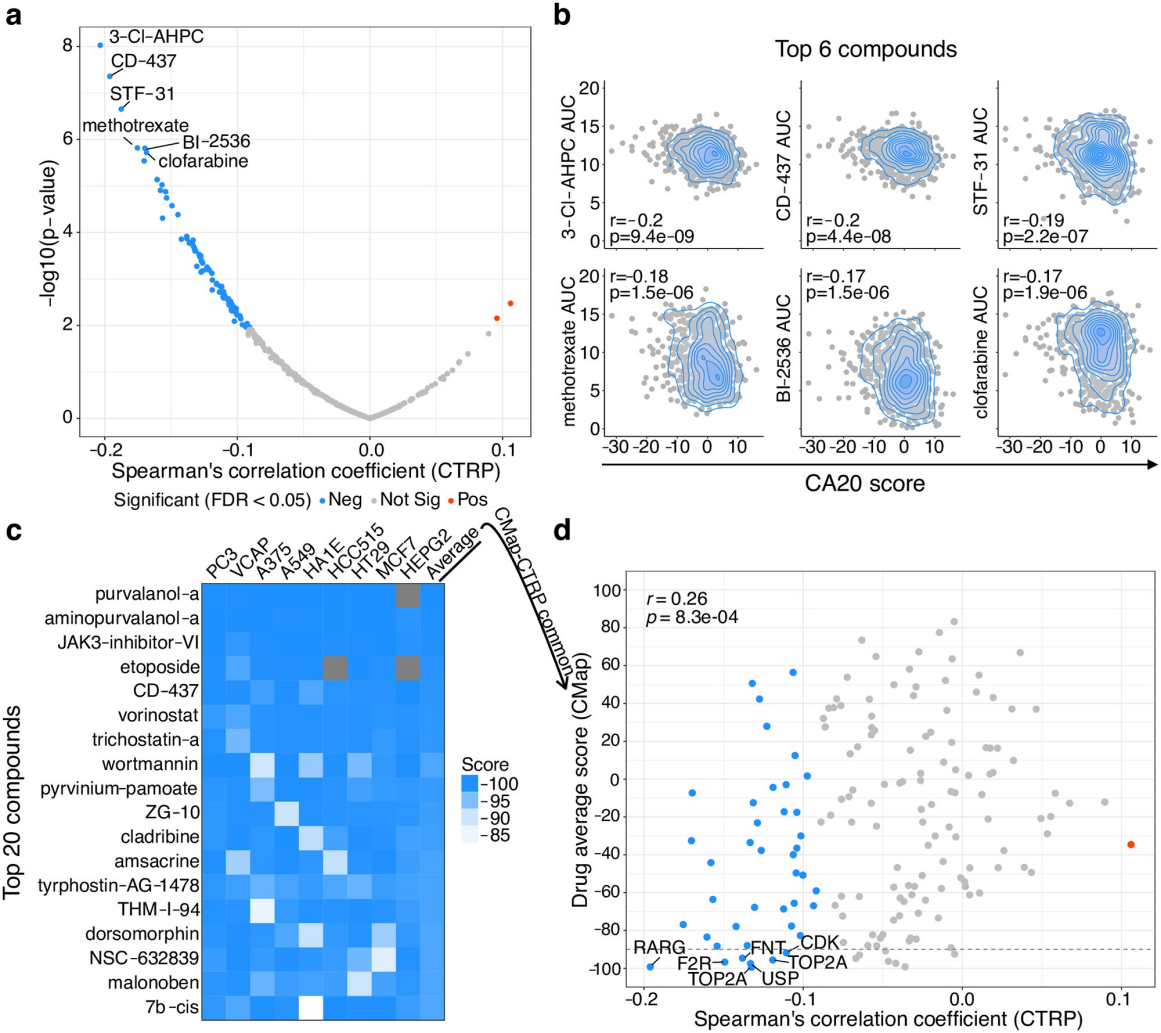
Identification of compounds that selectively kill cancer cells with high CA20. (**a**) Compounds with selective activity on cell lines with high or low CA20 score. The volcano plot shows the results of Spearman’s correlation analyses between CA20 scores and compound Area Under the dose-response Curve (AUC) across Cancer Therapeutics Response Portal (CTRP) human cancer cell lines. Note that lower AUC means higher drug activity. The compounds whose activity was associated with high and low CA20 (FDR < 0.05) are represented in blue and red, respectively. The top 6 compounds are highlighted. (**b**) Top 6 compounds targeting cells with higher CA20 score. Smooth scatter plots showing correlation between CA20 score and compound AUC across CTRP cell lines for the top 6 compounds from (a). Spearman’s correlation coefficients, r, and respective p-values are shown. (**c**) Compounds that down-regulate the CA20 gene set. Heatmap of CMap’s drug score, ranging from 100 (maximum CA20 up-regulation) to -100 (maximum CA20 down-regulation) per cell line. Drug average score (last column) is the mean of drug scores across cell lines. The 20 compounds with the lowest drug average score are shown and ranked accordingly. Tissue of origin of human cancer cell lines: PC3: prostate; VCAP: prostate; A375: melanoma; A549: lung; HA1E: kidney; HCC515: lung; HT29: colon; MCF7: breast; HEPG2: liver. (**d**) Compounds selectively targeting cells with higher CA20 also down-regulate these genes. Scatter plot showing correlation between CTRP’s Spearman’s correlation coefficient from (a) and CMap’s drug average score from (c) for the 164 compounds tested in both datasets (Spearman’s correlation coefficient, r = 0.26, p-value = 8.3e-4). Points are coloured as in (a) and the predicted protein targets of compounds with both significant Spearman’s correlations and drug average score lower than -90 are shown.

CA is a hallmark of cancer cells and hence an appealing target in cancer therapy. In order to identify compounds that could target cancer cells with such abnormality, we have employed CA20 to estimate relative CA levels in 823 human cancer cell lines from the Cancer Therapeutics Response Portal (CTRP) [58] (S12 Table), for which both transcriptomic and drug-sensitivity profiles are publicly available. Correlation analyses between CA20 and drug-sensitivity (in Area Under the dose-response Curve, AUC) for 354 compounds revealed 81 negatively correlated with CA20 (FDR < 0.05, Spearman’s correlation; Fig 6a and S13 Table), i.e. higher CA20 was associated with lower drug AUC and, therefore, higher drug activity. The enrichment of negative correlations (S14 Fig) may reflect the bias for cancer-targeting compounds in CTRP. These results suggest several candidate compounds to selectively kill cancer cells with CA, such as 3-CI-AHPC, CD-437, STF-31, methotrexate, BI-2536 and clofarabine (Fig 6b). The first three are probes, methotrexate and clofarabine are U.S. Food and Drug Administration (FDA)-approved drugs for several cancer types (https://www.cancer.gov/about-cancer/treatment/drugs/methotrexate) and paediatric acute lymphoblastic leukemia (https://www.cancer.gov/about-cancer/treatment/drugs/fda-clofarabine), respectively. Interestingly, BI-2536 has been in clinical trials for several solid and liquid tumours (https://clinicaltrials.gov/ct2/results?cond=&term=bi+2536&cntry=&state=&city=&dist) and is an inhibitor of polo-like kinase 1 (PLK1), whose inhibition has already been associated with CA suppression [59,60].

Complementarily, we mined the CMap database to identify compounds that could impact the CA20 score and therefore putatively reduce/increase CA levels. We calculated the impact of 2,837 compounds on the CA20 transcriptomic levels in human cancer cell lines (S14 Table) and identified some whose activity drove CA20 up-regulation (putative CA promoters; S15 Fig), such as VEGFR2-kinase-inhibitor-IV, dienestrol (oestrogen receptor agonist) and sulforaphane (anticancer agent in clinical trials for Bladder, Breast, Lung and Prostate cancers; https://clinicaltrials.gov/ct2/results?cond=sulforaphane&Search=Apply&recrs=d&age_v=&gndr=&type=&rslt=). We also identified compounds that down-regulated CA20, such as two CDK inhibitors (purvalanol-a and aminopurvalanol-a), JAK3-inhibitor-VI, etoposide (topoisomerase and cell cycle inhibitor) and CD-437 (agonist of RARG, retinoic acid receptor gamma; Fig 6c).

For the 164 drugs tested in both datasets, we observed a positive correlation between their CA20/sensitivity correlations in CTRP and their CMap scores (Spearman’s correlation coefficient, r = 0.26, p-value = 8.3e-4; Fig 6d), indicating that drugs selectively targeting cells with higher CA20 are reducing the expression of these genes, possibly by killing the abnormal cells in the tumour cell population. These complementary approaches uncovered RARG’s agonist CD-437 as the strongest candidate for targeting CA. Moreover, drugs targeting coagulation factor II (F2R), farnesyltransferase (FNTA and FNTB), ubiquitin isopeptidases (USP13 and USP5), DNA topoisomerase II alpha (TOP2A) and cyclin-dependent kinases (CDKs) are also promising candidates (Fig 6d). Given cell proliferation’s association with CA20 (S1a Fig), we have tested the association between its estimated rates across TCGA primary tumour samples and the expression of the 164 compounds’ predicted target genes (merging this information from the CTRP (S13 Table) and CMap datasets (S14 Table)), using linear regression analyses with cohort as additional covariate (S2 Table). The resultant coefficients (S15 Table) are not correlated with CMap’s average scores of the respective compounds (Spearman’s correlation coefficient, r = 0.016, p-value = 0.84; S16a Fig), but are correlated with their CTRP’s Spearman correlation coefficients (Spearman’s correlation coefficient, r = -0.26, p-value = 9e-04; S16b Fig), i.e. compounds selective for cells with high CA20 are predicted to target genes positively associated with proliferation in TCGA tumour samples. Nevertheless, predicted target genes of several compound candidates from our analyses do not show strong association with proliferation (S16c and S16d Fig). These results need to be considered when prioritizing candidate compounds for further experiments aiming to target cancer cells through CA.

## Discussion

CA is known to promote tumourigenesis but its molecular role therein remains elusive and, although it is also suggested to be a promising target for cancer therapy, CA’s prevalence in different types of cancer and therapeutic value in the clinic are still pretty much unprobed. Using the CA20 signature and TCGA RNA-seq data, we characterise the landscape of CA-associated gene expression in a broad range of cancer types, thereby demonstrating the potential of using gene expression-based signatures in multi-omic and clinical data integrative approaches to investigate the biological and medical relevance of their respective cellular and molecular processes.

Despite the lack of a full direct experimental validation of CA20 as a surrogate of CA levels, our observations are very consistent with known CA’s features, namely CA20’s upregulation in cancer [7] and in basal-like breast tumours [26], and its association with the knock-down of centriole duplication factors, genomic instability [11], loss of p53 [6,7,35] and pRB [44], hypoxia [54,55] and worse patient’s prognosis [7]. In addition, we found that luminal B breast tumours have higher prevalence of CA than luminal A ones, concordantly with the observed differences in the CA20 score between the two molecular subtypes in two independent cohorts. Finally, we have analysed two transcriptomic datasets of multiciliogenesis, where cells escape centriole number regulation to generate hundreds of centrioles during differentiation [61], and found that CA20 increases during the centriole overduplication stage, resuming basal levels afterwards (S17 Fig), suggesting CA20 as a marker of active amplification. These observations vouch for the present proof-of-concept study to pave the way for more in-depth and bona fide findings when CA’s transcriptomic signature is experimentally refined. Moreover, here we already propose novel hypotheses that will trigger studies aiming at a more comprehensive understanding of the role of CA in cancer.

We observed higher CA-associated gene expression in cancer samples of squamous cell origin than in adenocarcinomas, suggesting that their different cell types of origin can have different CA’s prevalence and/or ways to cope with this abnormality. Previous work has indeed shown that CA triggers spontaneous squamous cell carcinomas, lymphomas and sarcomas, but not adenocarcinomas, in mice [11]. We also show that breast invasive carcinoma samples have high variability on CA20, concordantly with previous observations [7,25], that is related to their distinct clinical and molecular features. We had recently shown that basal-like breast carcinomas have higher CA than luminal tumours [26], but here we report for the first time an upregulation of CA-associated genes in tumours from both invasive ductal histologic subtype and luminal B molecular subtype. We validated the CA20-based predictions by quantitatively analysing centrosome numbers in human breast carcinoma samples, where we found that indeed CA is more prevalent in luminal B than luminal A tumours, providing a novel insight into the differences between these two hormone-receptor positive molecular subtypes. Given the limited number of luminal B samples in our cohort, more extensive analyses are necessary to confirm this association. Our data show that centrosome amplification is associated with breast cancer clinical features and endorses the potential of using a gene-expression-based signature for patient stratification.

CA-associated gene expression upregulation is positively correlated with different types of genomic instability, like aneuploidy, mutation burden, CNA and tumour heterogeneity. In particular, CA20 is more strongly associated with chromosomal deletions than amplifications, independently of *TP53* mutations. We speculate that this association may be due to the impact of CA in cellular genomic stability having non-random genomic “hot spots”. In fact, through a more detailed analysis, we found an association with alterations in specific chromosomal arms, that may be due to the localisation of genes encoding for regulators of CA20 genes therein and/or to those arms’ higher susceptibility to the genomic instability triggered by centrosome abnormalities. The latter is supported by recent work showing that human chromosome mis-segregation is not random and can be biased by inherent properties of individual chromosomes [62], and also by our observation that normal samples whose matched tumours lost 5q or 16p have higher CA20 predictive of those deletions (S6 Fig). Moreover, we characterised the DNA mutation spectrum associated with CA20 and found it to be enriched in C>T mutations, a signature characteristic of ageing, with which centrosome aberrations have also been associated [63–67]. Genes whose mutations are associated with CA20 are enriched in cancer driver genes, and particularly in Wnt/β-catenin signalling. Wnt/β-catenin signalling components interact with the centrosome [68] and a previous study has demonstrated that mutant β-catenin induces centrosome aberrations in normal epithelial cells and is required for CA in cancer cells [69]. Our results extend this previous association to human cancer samples, suggesting mutations in β-catenin might contribute to the observed CA in cancer. Finally, we show the usefulness of a novel approach whereby we integrated information on genes whose somatic mutations are associated with CA20 in TCGA tumour samples with the impact of their knock-downs on the CA20 expression in human cancer cell lines, aiming at unveiling candidate molecular players in CA in cancer.

Concordantly with previous work on CA [7], we observed that high CA20 is associated with poor patient’s survival in several cancer types. Furthermore, we found a positive correlation between CA20 and hypoxic levels in glioblastoma multiforme that is particularly interesting, due to its highly hypoxic microenvironment and HIF-1α levels [70], also shown to enhance migration and invasion of its tumour cells [71,72]. Given the observed association between CA and invasion of tumour cells [15,17], an exciting hypothesis is hypoxia-induced invasion being mediated through CA. When looking at the tumour cellular composition, we found that tumours with high CA20 have lower stromal and immune cell infiltration, although the latter is not independent of tumour genomic instability and proliferation rate. Detailed studies aiming to decouple these effects could provide relevant molecular insights when considering immunotherapy, alone or in combination with genotoxic and/or anti-proliferative therapeutic approaches. Moreover, by pioneering the integration of drug sensitivity with drug perturbation profiles in human cancer cell lines, we identify candidate compounds for selectively targeting cancer cells exhibiting transcriptomic evidence for CA. These compounds could be particularly useful in the treatment of cancer types we identified as having high CA and to whose current therapy patients respond poorly. For instance, their potential in specifically targeting basal-like and luminal B breast tumours could be assessed by taking advantage of resources like patient-derived tumour xenografts [73]. The observed ability of cells carrying extra centrosomes to manipulate the surrounding tumour cells and promote their invasiveness [15,17] suggests that targeting the former may be clinically more impactful. Given CA’s cancer-specificity, the compounds identified herein could underlie the development of novel targeted cancer therapeutic options.

## Methods

### Ethics statement

The study with human samples was conducted under the national regulative law for the handling of biological specimens from tumour banks, with samples being exclusively used for research purposes in retrospective studies, and was approved by the ethics committee of the Hospital Xeral-Cies, Vigo, Spain. Informed consent was obtained from all human participants.

### TCGA dataset

Publicly available RNAseqV2 (quantified through RNA-seq by Expectation Maximization) [74] and clinical data for 9,721 tumour and 725 matched-normal samples from The Cancer Genome Atlas (TCGA; https://cancergenome.nih.gov/) were downloaded from Firebrowse (http://firebrowse.org/). Gene expression (read counts) data were quantile-normalized using voom [75]. For each sample, the CA20 score was calculated as the sum of the across-sample (including both tumours and matched-normal samples) normalized (log2 median-centred) expression levels of the CA20 published signature genes [23]: *AURKA*, *CCNA2*, *CCND1*, *CCNE2*, *CDK1*, *CEP63*, *CEP152*, *E2F1*, *E2F2*, *LMO4*, *MDM2*, *MYCN*, *NDRG1*, *NEK2*, *PIN1*, *PLK1*, *PLK4*, *SASS6*, *STIL* and *TUBG1* (Fig 1a).

Predicted proliferation rates of each TCGA tumour sample were retrieved from [24] (n = 9,568). Whole genome doubling (corresponding to 0, 1 and ≥ 2 genome doubling events in the clonal evolution of the cancer), aneuploidy (both aneuploidy score—number of altered chromosome arms—and alterations per chromosome arm) and mutation burden characterizations were retrieved from [34] (n = 9,166). Since the chromosomal arm status was not available for TCGA normal samples, we have selected only those with no CNA in the chromosomal arms tested, to make sure they are intact. CNA (n = 8,879; copy number levels were derived with the GISTIC algorithm [76] and considered as CNA if having a score lower than -1 (loss) or higher than 1 (gain)) and mutation (n = 7,120; including classification as silent, missense, splice site or nonsense ones) processed data were downloaded from Firebrowse (http://firebrowse.org/). Mutations were classified as likely pathogenic and pathogenic based on ClinVar database’s (https://www.ncbi.nlm.nih.gov/clinvar/) variant summary annotation (ftp://ftp.ncbi.nlm.nih.gov/pub/clinvar/tab_delimited/variant_summary.txt.gz; accessed in November 12^th^ 2018), and 5,601 likely driver mutations were obtained from the Cancer Genome Interpreter (https://www.cancergenomeinterpreter.org/mutations; accessed in November 12^th^ 2018) [45]. The list of 299 cancer driver genes was retrieved from [43]. Intra-tumour heterogeneity data, measured by the number of clones per sample, were retrieved from [77] (n = 1,080). The mutational signature profiles were retrieved from mSignatureDB [48] (n = 9,004). The predicted fraction of stromal (stromal score) and immune (immune score) cells in TCGA tumour samples (n = 2,463) was retrieved from [78]. We used the scores calculated based on RNASeqV2 expression levels. Importantly, no CA20 gene was used by the authors to infer those cell proportions [78].

TCGA tumour samples were analysed for hypoxic status based on expression of 95 genes included in the hypoxia 99-metagene signature [56]. The four missing genes are three (*LOC149464*, *LOC56901* and *TIMM23*) for which expression levels were not available and *NDRG1*, excluded for being part of the CA20 gene signature. The hypoxia score was calculated like the CA20 score.

Additional clinical information for TCGA breast tumour samples was retrieved from [27].

### METABRIC dataset

Normalized gene expression data for 1992 primary breast tumours and 144 normal breast tissue samples from the Molecular Taxonomy of Breast Cancer International Consortium (METABRIC) [28] were retrieved from European Genome-Phenome Archive (EGAC00001000484). Gene expression was profiled with Illumina HT-12 v3 microarrays, with probe-level intensity values being mean-summarised per gene. The CA20 score was calculated as for the TCGA dataset. Clinical information for the same samples was downloaded from cBioPortal (http://www.cbioportal.org/) [79].

### Analyses of CA in human breast carcinoma samples

Quantification of CA in breast cancer samples was performed as described in [26]. Briefly, formalin-fixed and paraffin-embedded human breast carcinoma samples were consecutively retrieved from the files of the Department of Pathology, Hospital Xeral-Cies, Vigo, Spain. This series comprises 29 luminal A, 3 luminal B, 3 HER2 and 13 basal-like tumours. Some of these samples had already been used in one of our recent studies [26]. The status of the oestrogen receptor (ER), progesterone receptor (PR), epidermal growth factor receptor 2 (HER2), antigen Ki67, and the basal markers epidermal growth factor receptor, cytokeratin 5, cytokeratin 14, P-cadherin and Vimentin was previously characterized for all tumour cases. According to their immunoprofile, breast tumour samples were classified as luminal A (ER+, PR+, HER2− and Ki67−), luminal B (ER+, PR+, HER2 overexpressing or Ki67+), HER2 (ER-, PR-, HER2 overexpressing) or basal-like carcinomas (ER−, PR−, HER2−, basal marker+). Representative tumour areas were carefully selected and at least two tissue cores (0.6 mm in diameter) were deposited into a tissue microarray. This study was conducted under the national regulative law for the handling of biological specimens from tumour banks, with samples being exclusively used for research purposes in retrospective studies. Informed consent was obtained from all human participants.

For immunofluorescence staining, 3 μm-thick tissue sections were deparaffinised in Clear-Rite-3 (Thermo Scientific, USA, CA) and rehydrated using a series of solutions with decreasing concentrations of ethanol. High temperature (98 °C, 60 min) antigenic retrieval with Tris-EDTA pH = 9.0 (LeicaBio systems, UK) was performed, followed by incubation with UltraVision protein block (Thermo Scientific) for 30 min at room temperature. The slides were, afterwards, incubated with mouse anti-GT335 (1/800 dilution, Adipogen Ref. AG- 20B-0020-C100) and rabbit anti-pericentrin (1/250 dilution, Abcam AB4448) in UltraAb diluent (Thermo Scientific) overnight at 4 °C. The sections were then washed three times, 5 min per wash, with 1× PBS + 0.02% Tween20 before a 1 h room temperature incubation with the secondary antibodies, anti-IgG rabbit coupled to Alexa 488 and anti-IgG mouse coupled to Alexa-594 (Invitrogen), diluted at 1/500 in PBS. Finally, sections were washed extensively with 1× PBS + 0.02% Tween20 and then counterstained and mounted with Vectashield containing DAPI (VectorLabs, CA, USA).

Imaging was performed on a Zeiss Imager Z1 inverted microscope, equipped with an AxioCam MRm camera (Zeiss) and ApoTome (Zeiss), using the ×100 1.4 NA Oil immersion objective. Images were taken as Z-stacks in a range of 10–14 μm, with a distance between planes of 0.3 μm, and were deconvolved with AxioVision 4.8.1 software (Zeiss). Only the structures positive for GT335 (centriolar marker) and pericentrin (PCM marker) were analysed and scored. Between 5 and 107 cells were analysed for each patient and cells with more than 4 centrioles were considered as having CA (S4 Table).

### CTRP dataset

Normalized gene-level expression and drug sensitivity (n = 481 compounds) data for 823 human cancer cell lines from the Cancer Therapeutics Response Portal (CTRP) v2 were retrieved from [58]. The CA20 score was calculated as for the aforementioned datasets. Compounds with more than 20% of missing data (n = 127) were removed from the analyses. Area Under the dose-response Curve (AUC) was used as the metric of cell line’s drug sensitivity, measured over a 16-point concentration range. Note that lower AUC means higher drug activity.

### Connectivity Map dataset

The Connectivity Map (CMap) database of signatures [49] was interrogated using CA20 genes as an individual query in the CLUE L1000 tool (https://clue.io/l1000-query#individual, login required; CA20 genes were used as putative UP-regulated genes). For each of the 9 human cancer cell lines profiled within the Touchstone dataset (PC3, VCAP, A375, A549, HA1E, HCC515, HT29, MCF7 and HEPG2), a connectivity score was computed per perturbation (gene knock-down, gene overexpression, small molecule administration) [49], reflecting its effect on the expression of CA20 genes (except for *SASS6*, not profiled in this dataset). We calculated an average connectivity score per perturbation by averaging the 9 cell lines’ connectivity scores in order to have a more robust connectivity score that can be used across different cell types and tissues. Two types of perturbations were analysed: 3,799 gene knock-downs and 2,837 compounds. The Broad compound ID was used to match the 164 compounds tested by CMap and CTRP, so that the results of the analyses of the two datasets could be combined.

### Multiciliogenesis datasets

Normalized gene expression data for adult mouse airway epithelial cells during multiciliogenesis (triplicates for three different time points: days 0, 2 and 4) was retrieved from [80] (GEO dataset accession GSE73331). The CA20 score was calculated as for the TCGA dataset.

The transcriptomic alterations between non-ciliating mouse tracheal epithelial cells and those undergoing differentiation, through transition to an air-liquid interface culture (ALI), and harvested at four (ALI+4) or twelve (ALI+12) days, were retrieved from [81]. Those probe-level transcriptomic alterations were mean-summarised per gene.

### Spearman’s correlation

Spearman’s correlations were performed using the *cor*.*test* R function (method = ‘‘spearman”) [82]. The difference between two Spearman’s correlations was tested using the *paired*.*r* function from R package *psych* [83].

### Unpaired two-sample statistical analyses

Wilcoxon rank-sum tests were performed using the *wilcox*.*test* R function [82].

### Linear regression analyses

Multiple linear regression modelling was implemented using the *lm* function from R package *limma* [84]. Covariate collinearity was tested using the *corvif* function from [85], in which all covariates had a variance inflation factor below 2. All equations and respective statistics are shown in S2 Table.

We have normalised the genomic instability covariates using z-scores (number of standard deviations from the mean) to account for differences in the prevalence of aneuploidy, mutation burden, CNA and number of clones per cohort.

### Fligner-Killeen test of homogeneity of variances

Fligner-Killeen test was implemented using the *fligner*.*test* R function [82].

### Test of equal proportions

Proportions tests were performed using the *prop*.*test* R function [82].

### Two-way analysis of variance (ANOVA)

Two-way ANOVA was done using the *aov* R function [82].

### Hierarchical clustering analyses

Unsupervised hierarchical clustering of the multiple linear regression results per cancer type was performed using the *heatmap*.*2* function from R package *gplots* [86].

### Gene Set Enrichment Analyses

Genes ranked according to the knock-down connectivity score were analysed for pathway enrichment using Gene Set Enrichment Analysis [41,42] with default parameters. We used a list of 299 cancer driver genes from [43], a manually curated list of centriole duplication factors (93 genes, including 10 from the CA20 signature; S10 Table), gene sets retrieved from the KEGG pathway database (https://www.kegg.jp/) and the MSigDB’s Hallmark Gene Sets library [50]. Those with a False Discovery Rate (FDR) lower than 5% were considered significant.

### Survival analyses

Dividing patients into two subgroups by CA20 median value, the significance of differences in prognostic was estimated using Kaplan−Meier plots and log-rank tests, per cancer type, through R package *survival* [87].

### Q-Q plot of p-values

To calculate the expected Spearman’s correlation coefficients and p-values used in the quantile-quantile (Q-Q) plot (S14 Fig), we permutated 1000 times the drug-sensitivity (in AUC) of all compounds across cell lines and, for each permutated dataset, we calculated the respective CA20-AUC Spearman’s correlations. The expected values were obtained by median-summarizing the ranked 1000 permutations’ results.

#### Code availability

All the core code generated and used in this study is available on GitHub (https://github.com/bernardo-de-almeida/PanCancer_CentrosomeAmplification).

## Supporting information

S1 Fig Pan-cancer analyses of centrosome amplification-associated gene expression. (**a**) CA20 is correlated with proliferation rate. Smooth scatter plots showing correlation between CA20 score and predicted proliferation rate [1/h] across TCGA tumour samples (Spearman’s correlation coefficient, r = 0.4, p-value < 2.2e-16). (**b**) CA20 score distribution across different types of kidney, brain, melanoma and lung cancers. Black points and lines represent the median +/- upper/lower quartiles. Number of samples used in each violin is shown within brackets. **** p-value < 0.0001 and n.s. non-significant (Wilcoxon rank-sum test). (**c**) CA20 score distribution between adenocarcinoma and squamous cell carcinomas within cervical (CESC) and oesophageal (ESCA) cancer types. Black points and lines represent the median +/- upper/lower quartiles. Number of samples used in each violin is shown within brackets. ** p-value < 0.01 and *** p-value < 0.001 (Wilcoxon rank-sum test). CESC: cervical squamous cell carcinoma and endocervical adenocarcinoma; COADREAD: colon and rectum adenocarcinoma; ESCA: oesophageal carcinoma; GBM: glioblastoma multiforme; HNSC: head and neck squamous cell carcinoma; KICH: kidney chromophobe; KIRC: kidney renal clear cell carcinoma; KIRP: kidney renal papillary cell carcinoma; LGG: low-grade glioma; LUAD: lung adenocarcinoma; LUSC: lung squamous cell carcinoma; OV: ovarian serous cystadenocarcinoma; PAAD: pancreatic adenocarcinoma; PRAD: prostate adenocarcinoma; SKCM: skin cutaneous melanoma; STAD: stomach adenocarcinoma; UVM: uveal melanoma.
(PDF)

S2 Fig CA20 is associated with different breast cancer clinical and molecular features. (**a-c**) CA20 score distribution per (a) histological and (b) PAM50 molecular subtype, and (c) tumour stage for TCGA breast cancer samples. For each category, samples were divided in low and high proliferation groups based on median predicted proliferation rate. Only samples with proliferation information were used. * p-value < 0.05, ** p-value < 0.01, *** p-value < 0.001, **** p-value < 0.0001 and n.s. non-significant (Wilcoxon rank-sum test). (**d-h**) CA20 score distribution between breast tumour histological subtypes grouped by triple-negative (TNBC) status (**d**,**f**), tumour stage (**e**,**g**), or integrative clusters (**h**, only for METABRIC samples) for (**d**,**e**) TCGA breast cancer and (**f**-**h**) METABRIC samples. Black points and lines represent the median +/- upper/lower quartiles. * p-value < 0.05, ** p-value < 0.01, **** p-value < 0.0001 and n.s. non-significant (Wilcoxon rank-sum test).
(PDF)

S3 Fig Luminal B and basal-like human breast carcinomas display higher levels of centrosome amplification. Distribution of the number of centrioles per cell observed in breast tumours from the different PAM50 molecular subtypes. Violin plots were created based on segments connecting frequencies at each integer (from 1 to 14 centrioles per cell), given that centriole number is a discrete variable. The number of cells analysed in the study, for each molecular subtype, is shown. ** p-value < 0.01, **** p-value < 0.0001 and n.s. non-significant (Wilcoxon rank-sum test).
(PDF)

S4 Fig CA20 is strongly associated with chromosomal deletions independently of *TP53* mutations. (**a** and **b**) CA20 is associated with both chromosomal deletions and amplifications. Smooth scatter plots showing correlation between CA20 score and number of (**a**) amplifications and (**b**) deletions across TCGA tumour samples (Spearman’s correlation coefficient, r = 0.41 and 0.36, respectively, p-value < 2.2e-16 for both). (**c**) CA20 is more strongly associated with chromosomal deletions. Smooth scatter plot showing correlation between CA20 score and the significance of the difference between the proportion of both features per sample across TCGA tumour samples (Spearman’s correlation coefficient, r = -0.1, p-value < 2.2e-16). The Y-axis represents the log10 of p-value for proportion tests, with positive or negative sign if the sample has higher proportion of amplifications or deletions, respectively. (**d**) Significance of the difference between the proportion of amplifications and deletions per sample (from **c**) in all (n = 8,092), *TP53* wild-type (n = 6,292) or *TP53* mutated (n = 1,080) TCGA tumour samples divided in low and high CA20 groups (based on CA20’s median). Black points and lines represent the median +/- upper/lower quartiles. * p-value < 0.05 and **** p-value < 0.0001 (Wilcoxon rank-sum test). Interaction between CA20 group and *TP53* status was assessed by two-way ANOVA (p-value = 0.6). (**e**) Number of amplifications (red) and deletions (blue) in all (n = 8,092), *TP53* wild-type (n = 6,292) or *TP53* mutated (n = 1,080) TCGA tumour samples divided in low and high CA20 groups (based on CA20’s median). Black points and lines represent the median +/- upper/lower quartiles. **** p-value < 0.0001 (Wilcoxon rank-sum test).
(PDF)

S5 Fig CA20 is pan-cancer-widely associated with deletion of chromosome arm 5q. Box plots of CA20 score per alteration (deletion, none, or amplification) on chromosome arm 5q within samples from (**a**) the TCGA breast cancer cohort and (**b**) all other TCGA cohorts. **** p-value < 0.0001 (linear regression).
(PDF)

S6 Fig Higher CA20 levels in TCGA normal samples whose matched tumours have alterations in 5q and 16p chromosomal arms. Box plots of CA20 score of TCGA normal samples per alteration (deletion, none, or amplification) of their matched tumour samples on chromosomal arm (**a**) 5q (n = 297), (**b**) 16p (n = 566) and (**c**) 7p (n = 571). All normal samples used here have no CNA in the respective chromosomal arm. * p-value < 0.05 and ** p-value < 0.01 (Wilcoxon rank-sum test).
(PDF)

S7 Fig CA20 is associated with genomic instability features independently of cell proliferation. Scatter plots showing correlation between CA20 score and (**a**) aneuploidy score (measured as the total number of altered chromosome arms), (**b**) number of mutations per Mb, (**c**) number of CNAs and (**d**) clones per tumour across TCGA tumour samples divided in low and high proliferation groups (based on median predicted proliferation rate). Multivariate linear regression (CA20 ~ *β*_0_ + *β*_1_*feature + *β*_2_*proliferation group + *β*_3_*cohort) p-values for each genomic feature and respective regression lines are shown. Shades around linear regression lines represent their 95% confidence interval. Only samples with information for proliferation rates were used.
(PDF)

S8 Fig CA20 is associated with different types of mutations. (**a**-**d**) Smooth scatter plots showing correlation between CA20 score and number of (**a**) silent, (**b**) missense, (**c**) splice site and (**d**) nonsense somatic mutations per Mb across TCGA tumour samples (Spearman’s correlation coefficient, r = 0.42, 0.45, 0.29 and 0.39, respectively, p-value < 2.2e-16 for all). Y-axes are in log10 scale. Only samples with at least one mutation are shown. (**e**-**f**) Scatter plots showing correlation between CA20 score and number of likely pathogenic and pathogenic (as defined in ClinVar; see Methods for more details) mutations in (**e**) all diseases or (**f**) only in cancer across TCGA tumour samples (Spearman’s correlation coefficient, r = 0.18 and 0.1, respectively, p-value < 2.2e-16 and = 2.6e-05). Only samples with at least one mutation are shown. 5 outlier samples with more than 20 mutations (52, 39, 29, 25 and 24 mutations) in **e** were removed for better visualisation.
(PDF)

S9 Fig Distribution of genomic instability features on TCGA. (**a** and **b**) Comparison of the z-score distribution of aneuploidy score, number of mutations per Mb, number of CNAs and clones per tumour (**a**) between and (**b**) within the 12 TCGA cancer types used in multiple linear regression analyses of Fig 3g. Black points represent the median values. BLCA: bladder urothelial carcinoma; CESC: cervical squamous cell carcinoma and endocervical adenocarcinoma; GBM: glioblastoma multiforme; HNSC: head and neck squamous cell carcinoma; KIRC: kidney renal clear cell carcinoma; LGG: low-grade glioma; LUAD: lung adenocarcinoma; LUSC: lung squamous cell carcinoma; PRAD: prostate adenocarcinoma; SKCM: skin cutaneous melanoma; STAD: stomach adenocarcinoma; THCA: thyroid carcinoma.
(PDF)

S10 Fig Genes whose mutations are associated with CA20 are enriched in cancer driver genes, cancer-associated pathways and Wnt/β-catenin signalling. (**a**) Gene Set Enrichment Analysis (GSEA) of genes ranked by their -log10 of linear regression p-value (from Fig 4a) using a list of 299 cancer driver genes derived from [43]. The GSEA p-value is shown. (**b**) Driver mutations pan-cancer-wide associated with the CA20 score. The volcano plot shows the results of linear regression analyses comparing the CA20 score between wild-type samples and samples with driver mutations for 33 genes (at least 10 samples with driver mutations). Genes whose driver mutations are associated with higher and lower CA20 (FDR < 0.05) are represented in red and blue, respectively. Box plot of CA20 score per *TP53* mutation status (wild-type, with passenger, or with driver mutation) is shown. (**c**) GSEA of genes ranked by their -log10 of linear regression p-value (from Fig 4a) using KEGG pathways. The 15 significantly enriched (FDR < 0.05) pathways are shown and those cancer-associated are highlighted in red. Positively (red) and negatively (blue) enriched pathways, with a FDR lower than 5%, are shown. (**d**) GSEA plot for the bladder cancer pathway (from **c**). The GSEA p-value is shown. (**e**) GSEA of genes ranked by their -log10 of linear regression p-value (from Fig 4a) using MSigDB’s Hallmark Gene Sets. The top 10 gene sets are shown. Only the Wnt/β-catenin signalling gene set is significantly enriched (FDR < 0.05; dark grey). (**f**) GSEA plot for the Wnt/β-catenin signalling gene set (from **e**). The GSEA p-value is shown.
(PDF)

S11 Fig Mutational signatures pan-cancer-wide associated with CA20 score. Left: Significance of linear regression analyses (-log10 p-value) between CA20 and contribution of each mutational signatures. Positive and negative significant associations (FDR < 0.05) are coloured in red and blue, respectively. Right: Smooth scatter plots showing correlations between CA20 score and contribution of mutational signatures 3, 13 and 4 in breast invasive carcinoma (BRCA) and lung adenocarcinoma (LUAD). Mutational process associated with each signature (in parenthesis) and linear regression p-values are shown.
(PDF)

S12 Fig Patterns of mutational signatures 1 and 12. (**a** and **b**) Percentage of mutations attributed to each of the 96 substitutions, defined by the substitution class and sequence context immediately 5′ and 3′ to the mutated base, in mutational signatures (**a**) 1 and (**b**) 12. The probability bars for the six substitution classes are displayed in different colours. Images retrieved from https://cancer.sanger.ac.uk/cosmic/signatures.
(PDF)

S13 Fig CA20 is associated with hypoxia and stromal and immune cell infiltration. (**a**,**b**,**d**) Scatter plots showing correlation between CA20 score and (**a**) hypoxia, (**b**) stromal and (**d**) immune scores across TCGA tumour samples divided in low and high proliferation groups (based on median predicted proliferation rate). Multivariate linear regression (CA20 ~ *β*_0_ + *β*_1_*hypoxia score + *β*_2_*aneuploidy score + *β*_3_*mutation burden + *β*_4_*CNA + *β*_5_*clones per tumour + *β*_6_*proliferation rate + *β*_7_*cohort or CA20 ~ *β*_0_ + *β*_1_*stromal score + *β*_2_*immune score + *β*_3_*aneuploidy score + *β*_4_*mutation burden + *β*_5_*CNA + *β*_6_*clones per tumour + *β*_7_*proliferation rate + *β*_8_*cohort) p-values for each feature and respective regression lines are shown. Shades around linear regression lines represent their 95% confidence interval. Only samples with information for proliferation rates and genomic instability features were used. (**c**) Higher CA20 is associated with lower immune cell infiltration. Smooth scatter plot showing correlation between the CA20 and the immune scores across TCGA tumour samples (Spearman’s correlation coefficient, r = -0.34, p-value < 2.2e-16). (**e**) Higher CA20 is associated with lower immune cell infiltration in glioblastoma and higher infiltration in head and neck squamous cell carcinoma. Linear regression coefficients, representing the CA20 score dependence on the immune score, independently of genomic instability, across the TCGA cohorts with information for all covariates. Significant associations (FDR < 0.05) are coloured. BLCA: bladder urothelial carcinoma; GBM: glioblastoma multiforme; HNSC: head and neck squamous cell carcinoma; KIRC: kidney renal clear cell carcinoma; LUAD: lung adenocarcinoma.
(PDF)

S14 Fig Q-Q plot of CTRP CA20-AUC Spearman's correlation results. Quantile-quantile (Q-Q) plot of observed versus expected–log10 of Spearman’s correlation p-values between CA20 and drug-sensitivity (in AUC), with positive or negative sign if the correlation is positive or negative, respectively, across the Cancer Therapeutics Response Portal (CTRP) human cancer cell lines for 354 compounds. The solid line in the Q-Q plot indicates the distribution of compounds under the null hypothesis of no correlation. The compounds whose activity was associated with high and low CA20 (FDR < 0.05; Fig 6a) are represented in blue and red, respectively.
(PDF)

S15 Fig Compounds that up-regulate the CA20 gene set. Heatmap of CMap’s drug score, ranging from 100 (maximum CA20 up-regulation) to -100 (maximum CA20 down-regulation) per cell line. Drug average score (last column) is the mean of drug scores across cell lines. The 20 compounds with the highest drug average score are shown and ranked accordingly. Tissue of origin of human cancer cell lines: PC3: prostate; VCAP: prostate; A375: melanoma; A549: lung; HA1E: kidney; HCC515: lung; HT29: colon; MCF7: breast; HEPG2: liver.
(PDF)

S16 Fig Association between compounds’ targets and cell proliferation of TCGA samples. We used linear regression (gene expression ~ *β*_0_ + *β*_1_*proliferation rate + *β*_2_*cohort) to calculate the association between expression of each compound’s predicted target gene (we merged compound target annotations from the CTRP and CMap datasets) and proliferation rates across TCGA primary tumour samples (S15 Table). (**a** and **b**) Scatter plots showing correlations between linear regression coefficient and (**a**) CMap’s average scores or (**b**) CTRP’s Spearman correlation coefficients of the respective compounds (Spearman’s correlation coefficient, r = 0.016 and -0.26, p-value = 0.84 and 9e-04, respectively). (**c**) As in Fig 6d, but with compounds coloured by the linear regression coefficient of the predicted target gene (using the strongest association when a compound has more than one target gene). The two compounds with no annotated target gene are represented in grey. (**d**) Example for gene *RARG*. Smooth scatter plot showing correlation between *RARG* gene expression and predicted proliferation rates of TCGA primary tumour samples. The linear regression p-value is shown.
(PDF)

S17 Fig CA20 is a surrogate for centriole overduplication during multiciliogenesis. (**a**) CA20 increases from day 0 to day 2, and then decreases to day 4, during multiciliogenesis of adult mouse airway epithelial progenitors cultured in air-liquid interface (ALI). * p-value < 0.05 and ** p-value < 0.01 (Wilcoxon rank-sum test). Data from Mori et al., 2017. (**b**) Some CA20 genes (*PLK4*, *STIL*, *CEP152* and *SASS6*) are significantly upregulated only at the fourth day of multiciliogenesis. Significance of differential expression of CA20 genes between non-ciliating cells and cells undergoing multiciliogenesis harvested at four days (ALI +4), to enrich for genes involved in initial steps of centriole duplication, and twelve days (ALI+12), to enrich for genes expressed when cilia are mature [81]. The Y-axis represents the log10 of FDR-adjusted p-value for differential expression, with positive or negative sign if the sample has higher or lower expression than non-ciliating cells, respectively. Data from Hoh et al., 2012. (**c**) GSEA of the CA20 gene set on genes ranked by their signed log10 of FDR-adjusted p-value for differential expression, as in (b), between non-ciliating and ALI+4 or ALI+12 cells. GSEA p-values are shown.
(PDF)

S1 Table CA20 score across The Cancer Genome Atlas (TCGA) tumour and matched-normal samples. (TXT)

S2 Table Results of all linear regression analyses performed in this study, except those involving multiple testing, whose results are presented in individual supplementary S5, S7, S8 and S15 Tables. (XLSX)

S3 Table CA20 score across METABRIC tumour and normal samples. (TXT)

S4 Table CA levels in human breast carcinoma samples. Both the number of cells with different number of centrioles and the percentage of cells with CA (>4 centrioles) are shown.
(XLSX)

S5 Table Results of pan-cancer-wide linear regression analyses comparing CA20 score between samples with deletion or amplification of each chromosome arm. Related to Fig 3c.
(TXT)

S6 Table Data used in multiple linear regression analyses to identify independent associations between genomic instability features and CA20 across 1050 tumour samples (from 12 different cancer types). Related to Fig 3g.
(TXT)

S7 Table Results of pan-cancer-wide linear regression analyses comparing CA20 score between mutated and wild-type samples for 14,589 genes (mutated in at least 20 samples). Related to Fig 4a.
(TXT)

S8 Table Results of pan-cancer-wide linear regression analyses comparing CA20 score between driver mutated and wild-type samples for 33 genes (with driver mutations in at least 10 samples). Related to S10b Fig.
(TXT)

S9 Table Connectivity Map (CMap)’s knock-down scores on the CA20 gene set in human cancer cell lines for 3,799 genes. Related to Fig 4d.
(TXT)

S10 Table Manually curated list of centriole duplication factors. (TXT)

S11 Table Results of survival analyses across 31 TCGA cancer types with more than 40 samples. Related to Fig 5a.
(TXT)

S12 Table CA20 score across Cancer Therapeutics Response Portal human cancer cell lines. (TXT)

S13 Table Results of Spearman’s correlation analyses between CA20 scores and compound Area Under the dose-response Curve (AUC) across CTRP human cancer cell lines for 354 compounds. Related to Fig 6a and 6d.
(TXT)

S14 Table Connectivity Map (CMap)’s drug scores on the CA20 gene set in human cancer cell lines for 2,837 compounds. Related to Fig 6c and 6d.
(TXT)

S15 Table Results of linear regression analyses comparing the expression of compounds’ target genes from Fig 6d and proliferation rates across TCGA primary tumour samples. Related to S16 Fig.
(TXT)
