# Supplementary material for: Pan-cancer association of a centrosome amplification gene expression signature with genomic alterations and clinical outcome
Source: PLoS Comput Biol. 2019 Mar 11;15(3):e1006832. doi: 10.1371/journal.pcbi.1006832 (PMC6411098; doi:10.1371/journal.pcbi.1006832)
Supplement: S1 Fig — (a) CA20 is correlated with proliferation rate. Smooth scatter plots showing correlation between CA20 score and predicted proliferation rate [1/h] across TCGA tumour samples (Spearman’s correlation coefficient, r = 0.4, p-value < 2.2e-16). (b) CA20 score distribution across different types of kidney, brain, melanoma and lung cancers. Black points and lines represent the median +/- upper/lower quartiles. Number of samples used in each violin is shown within brackets. **** p-value < 0.0001 and n.s. non-significant (Wilcoxon rank-sum test). (c) CA20 score distribution between adenocarcinoma and squamous cell carcinomas within cervical (CESC) and oesophageal (ESCA) cancer types. Black points and lines represent the median +/- upper/lower quartiles. Number of samples used in each violin is shown within brackets. ** p-value < 0.01 and *** p-value < 0.001 (Wilcoxon rank-sum test). CESC: cervical squamous cell carcinoma and endocervical adenocarcinoma; COADREAD: colon and rectum adenocarcinoma; ESCA: oesophageal carcinoma; GBM: glioblastoma multiforme; HNSC: head and neck squamous cell carcinoma; KICH: kidney chromophobe; KIRC: kidney renal clear cell carcinoma; KIRP: kidney renal papillary cell carcinoma; LGG: low-grade glioma; LUAD: lung adenocarcinoma; LUSC: lung squamous cell carcinoma; OV: ovarian serous cystadenocarcinoma; PAAD: pancreatic adenocarcinoma; PRAD: prostate adenocarcinoma; SKCM: skin cutaneous melanoma; STAD: stomach adenocarcinoma; UVM: uveal melanoma. (PDF) [file pcbi.1006832.s001.pdf]

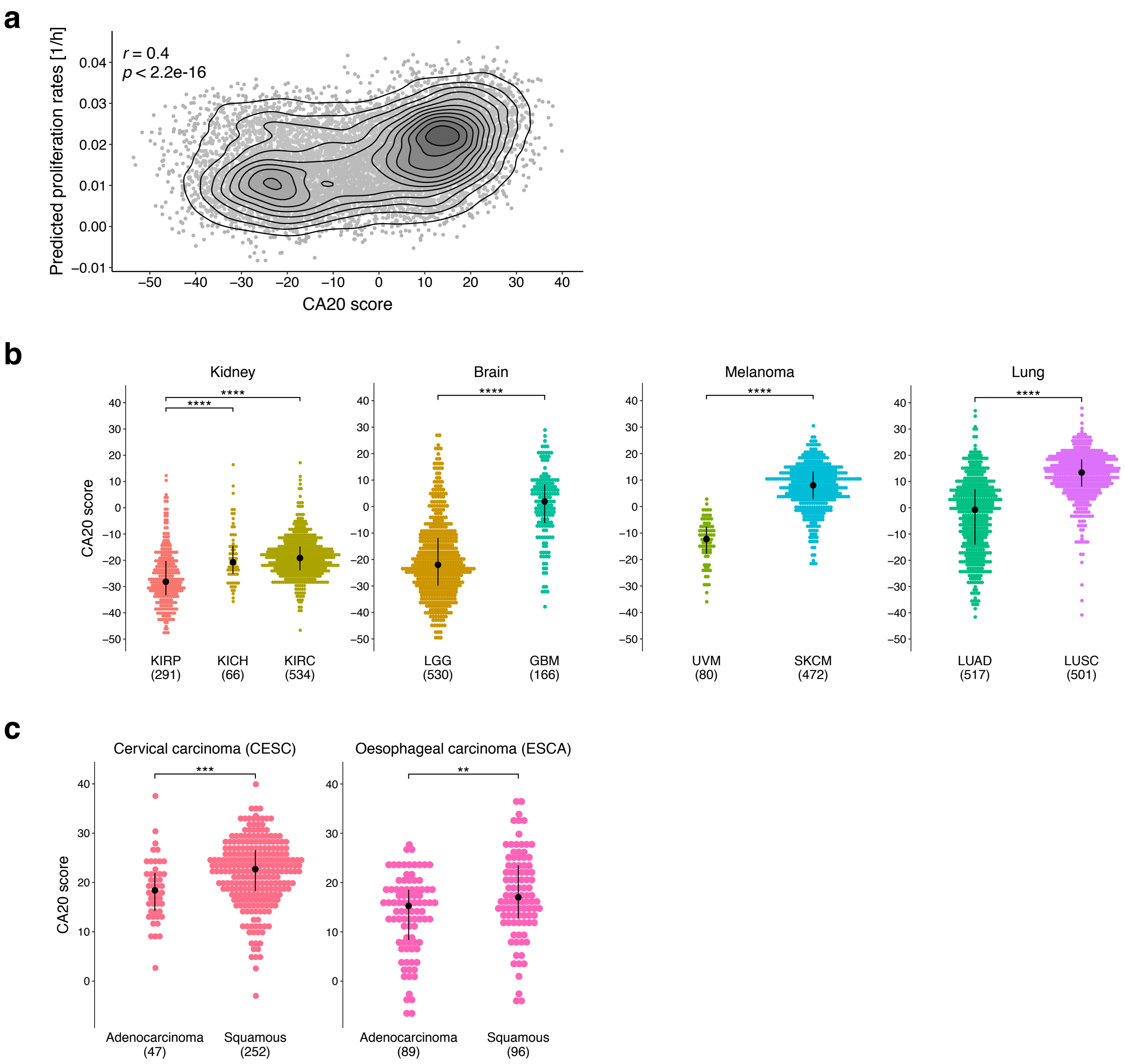

Supplementary Figure 1: Pan-cancer analyses of centrosome amplification-associated gene expression.
