# Supplementary material for: Pan-cancer association of a centrosome amplification gene expression signature with genomic alterations and clinical outcome
Source: PLoS Comput Biol. 2019 Mar 11;15(3):e1006832. doi: 10.1371/journal.pcbi.1006832 (PMC6411098; doi:10.1371/journal.pcbi.1006832)
Supplement: S2 Fig — (a-c) CA20 score distribution per (a) histological and (b) PAM50 molecular subtype, and (c) tumour stage for TCGA breast cancer samples. For each category, samples were divided in low and high proliferation groups based on median predicted proliferation rate. Only samples with proliferation information were used. * p-value < 0.05, ** p-value < 0.01, *** p-value < 0.001, **** p-value < 0.0001 and n.s. non-significant (Wilcoxon rank-sum test). (d-h) CA20 score distribution between breast tumour histological subtypes grouped by triple-negative (TNBC) status (d,f), tumour stage (e,g), or integrative clusters (h, only for METABRIC samples) for (d,e) TCGA breast cancer and (f-h) METABRIC samples. Black points and lines represent the median +/- upper/lower quartiles. * p-value < 0.05, ** p-value < 0.01, **** p-value < 0.0001 and n.s. non-significant (Wilcoxon rank-sum test). (PDF) [file pcbi.1006832.s002.pdf]

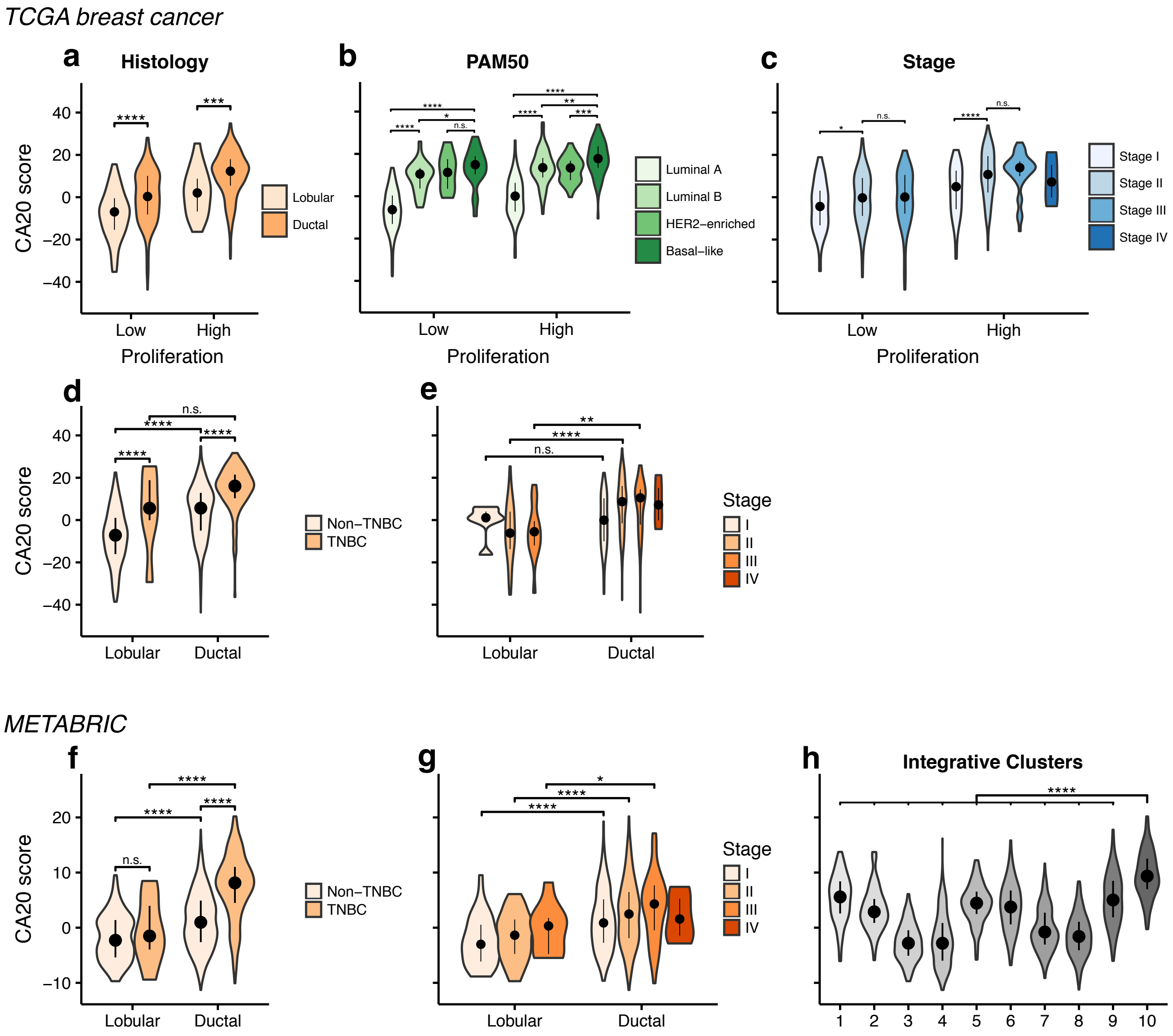

Supplementary Figure 2: CA20 is associated with different breast cancer clinical and molecular features.
