# Supplementary material for: Pan-cancer association of a centrosome amplification gene expression signature with genomic alterations and clinical outcome
Source: PLoS Comput Biol. 2019 Mar 11;15(3):e1006832. doi: 10.1371/journal.pcbi.1006832 (PMC6411098; doi:10.1371/journal.pcbi.1006832)
Supplement: S3 Fig — Distribution of the number of centrioles per cell observed in breast tumours from the different PAM50 molecular subtypes. Violin plots were created based on segments connecting frequencies at each integer (from 1 to 14 centrioles per cell), given that centriole number is a discrete variable. The number of cells analysed in the study, for each molecular subtype, is shown. ** p-value < 0.01, **** p-value < 0.0001 and n.s. non-significant (Wilcoxon rank-sum test). (PDF) [file pcbi.1006832.s003.pdf]

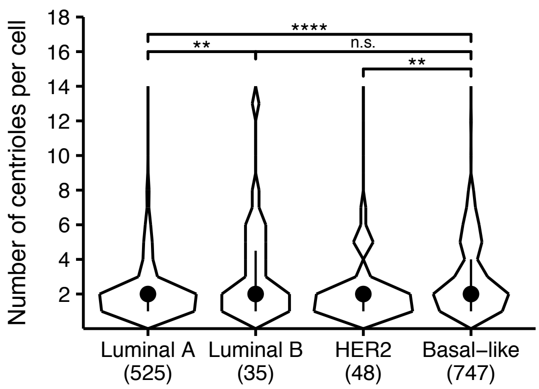

Supplementary Figure 3: Luminal B and basal-like human breast carcinomas display higher levels of centrosome amplification.
