# Supplementary material for: Pan-cancer association of a centrosome amplification gene expression signature with genomic alterations and clinical outcome
Source: PLoS Comput Biol. 2019 Mar 11;15(3):e1006832. doi: 10.1371/journal.pcbi.1006832 (PMC6411098; doi:10.1371/journal.pcbi.1006832)
Supplement: S4 Fig — (a and b) CA20 is associated with both chromosomal deletions and amplifications. Smooth scatter plots showing correlation between CA20 score and number of (a) amplifications and (b) deletions across TCGA tumour samples (Spearman’s correlation coefficient, r = 0.41 and 0.36, respectively, p-value < 2.2e-16 for both). (c) CA20 is more strongly associated with chromosomal deletions. Smooth scatter plot showing correlation between CA20 score and the significance of the difference between the proportion of both features per sample across TCGA tumour samples (Spearman’s correlation coefficient, r = -0.1, p-value < 2.2e-16). The Y-axis represents the log10 of p-value for proportion tests, with positive or negative sign if the sample has higher proportion of amplifications or deletions, respectively. (d) Significance of the difference between the proportion of amplifications and deletions per sample (from c) in all (n = 8,092), TP53 wild-type (n = 6,292) or TP53 mutated (n = 1,080) TCGA tumour samples divided in low and high CA20 groups (based on CA20’s median). Black points and lines represent the median +/- upper/lower quartiles. * p-value < 0.05 and **** p-value < 0.0001 (Wilcoxon rank-sum test). Interaction between CA20 group and TP53 status was assessed by two-way ANOVA (p-value = 0.6). (e) Number of amplifications (red) and deletions (blue) in all (n = 8,092), TP53 wild-type (n = 6,292) or TP53 mutated (n = 1,080) TCGA tumour samples divided in low and high CA20 groups (based on CA20’s median). Black points and lines represent the median +/- upper/lower quartiles. **** p-value < 0.0001 (Wilcoxon rank-sum test). (PDF) [file pcbi.1006832.s004.pdf]

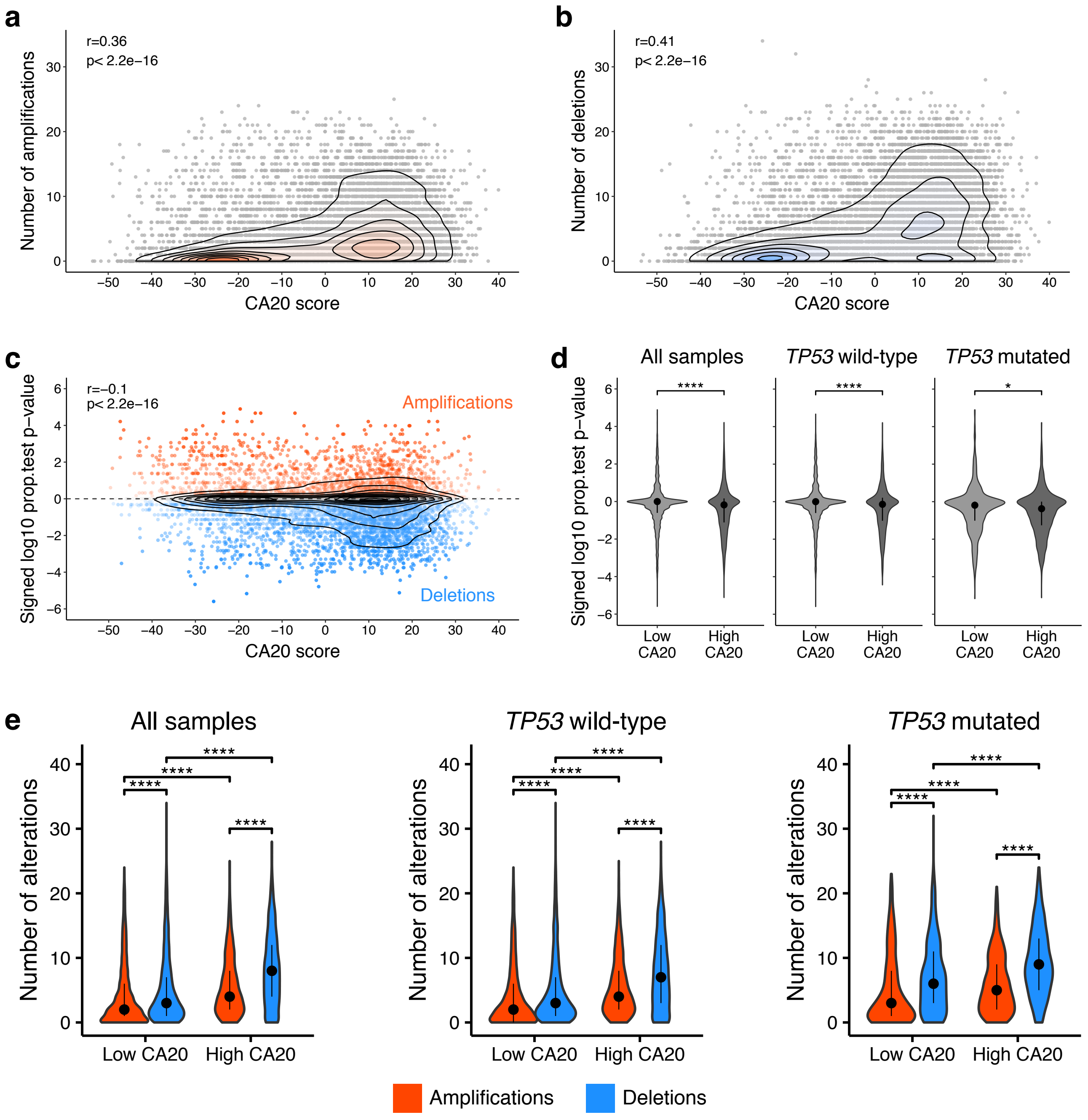

Supplementary Figure 4: CA20 is strongly associated with chromosomal deletions independently of *TP53* mutations.
