# Supplementary figures and images for: Pan-cancer association of a centrosome amplification gene expression signature with genomic alterations and clinical outcome
Source: PLoS Comput Biol. 2019 Mar 11;15(3):e1006832. doi: 10.1371/journal.pcbi.1006832 (PMC6411098; doi:10.1371/journal.pcbi.1006832)

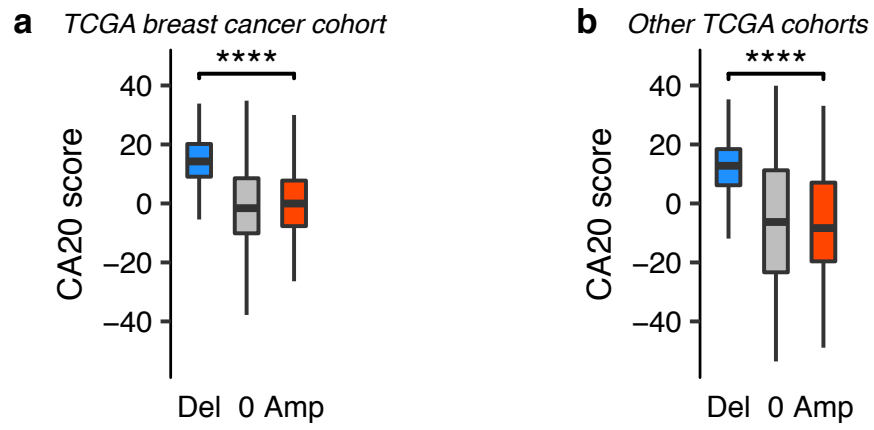

**Supplementary Figure 5:** CA20 is pan-cancer-widely associated with deletion of chromosome arm 5q.

Supplement: S5 Fig — Box plots of CA20 score per alteration (deletion, none, or amplification) on chromosome arm 5q within samples from (a) the TCGA breast cancer cohort and (b) all other TCGA cohorts. **** p-value < 0.0001 (linear regression). (PDF) [file pcbi.1006832.s005.pdf]

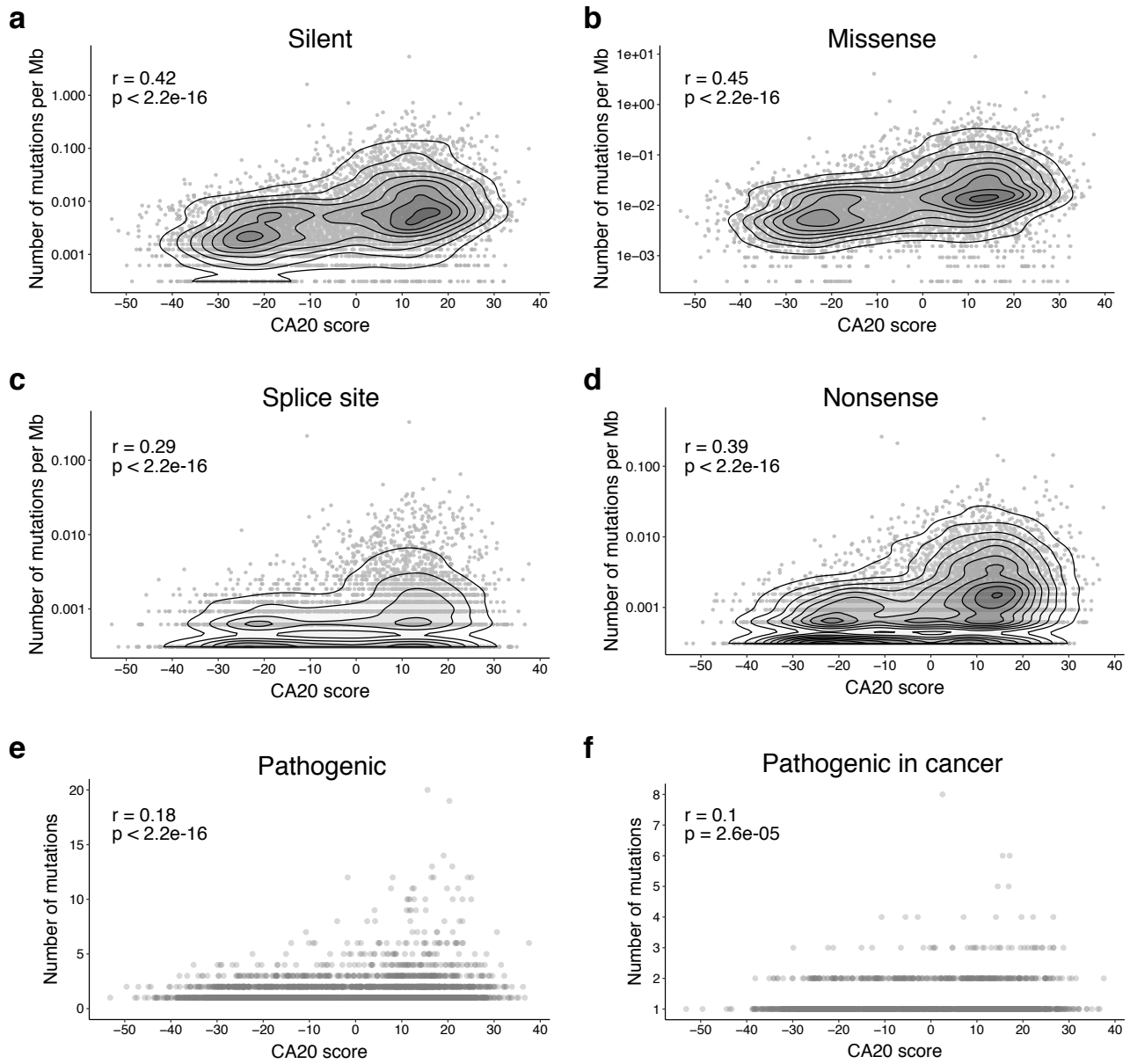

**Supplementary Figure 8: CA20 is associated with different types of mutations.**

Supplement: S8 Fig — (a-d) Smooth scatter plots showing correlation between CA20 score and number of (a) silent, (b) missense, (c) splice site and (d) nonsense somatic mutations per Mb across TCGA tumour samples (Spearman’s correlation coefficient, r = 0.42, 0.45, 0.29 and 0.39, respectively, p-value < 2.2e-16 for all). Y-axes are in log10 scale. Only samples with at least one mutation are shown. (e-f) Scatter plots showing correlation between CA20 score and number of likely pathogenic and pathogenic (as defined in ClinVar; see Methods for more details) mutations in (e) all diseases or (f) only in cancer across TCGA tumour samples (Spearman’s correlation coefficient, r = 0.18 and 0.1, respectively, p-value < 2.2e-16 and = 2.6e-05). Only samples with at least one mutation are shown. 5 outlier samples with more than 20 mutations (52, 39, 29, 25 and 24 mutations) in e were removed for better visualisation. (PDF) [file pcbi.1006832.s008.pdf]

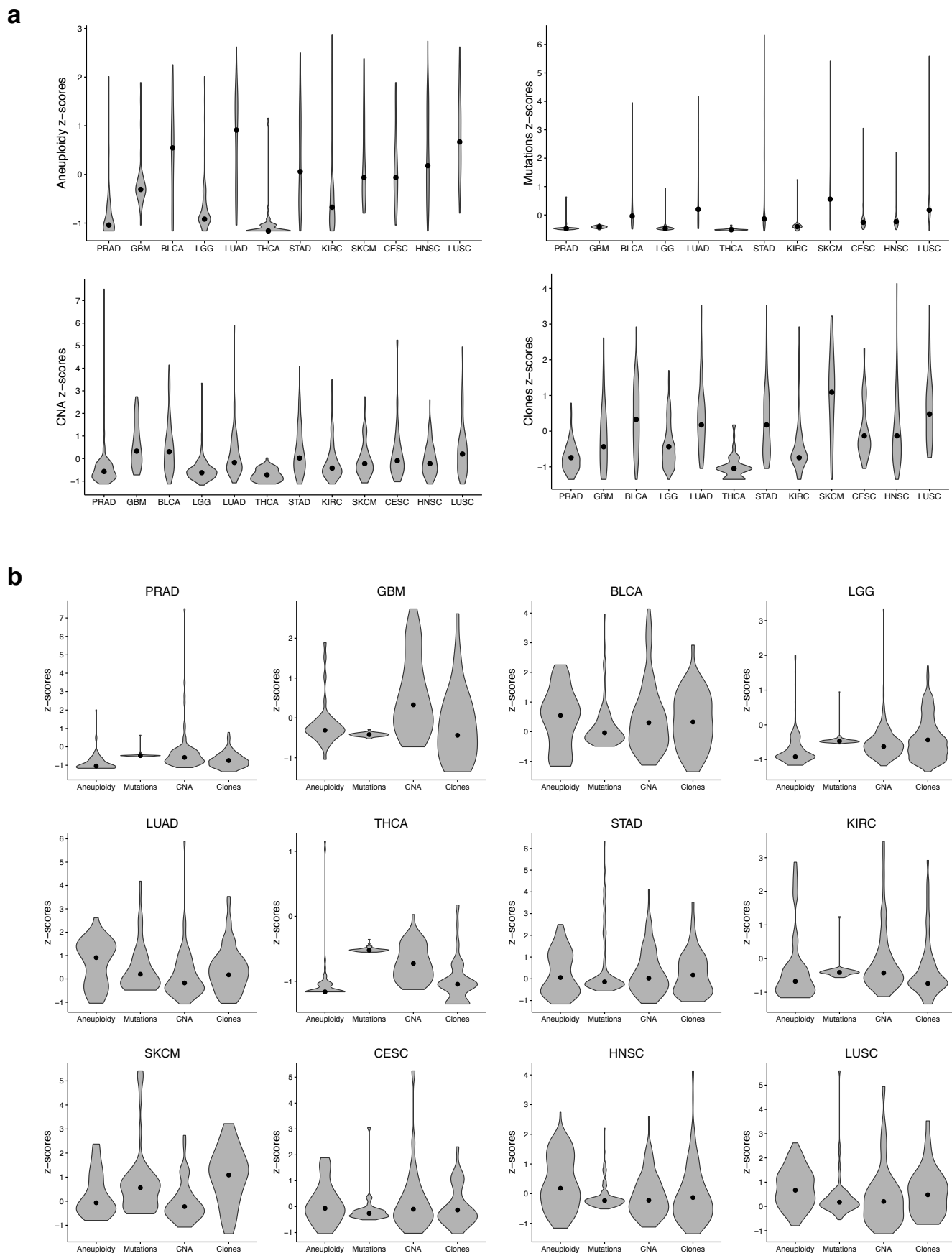

**Supplementary Figure 9: Distribution of genomic instability features on TCGA.**

Supplement: S9 Fig — (a and b) Comparison of the z-score distribution of aneuploidy score, number of mutations per Mb, number of CNAs and clones per tumour (a) between and (b) within the 12 TCGA cancer types used in multiple linear regression analyses of Fig 3g. Black points represent the median values. BLCA: bladder urothelial carcinoma; CESC: cervical squamous cell carcinoma and endocervical adenocarcinoma; GBM: glioblastoma multiforme; HNSC: head and neck squamous cell carcinoma; KIRC: kidney renal clear cell carcinoma; LGG: low-grade glioma; LUAD: lung adenocarcinoma; LUSC: lung squamous cell carcinoma; PRAD: prostate adenocarcinoma; SKCM: skin cutaneous melanoma; STAD: stomach adenocarcinoma; THCA: thyroid carcinoma. (PDF) [file pcbi.1006832.s009.pdf]

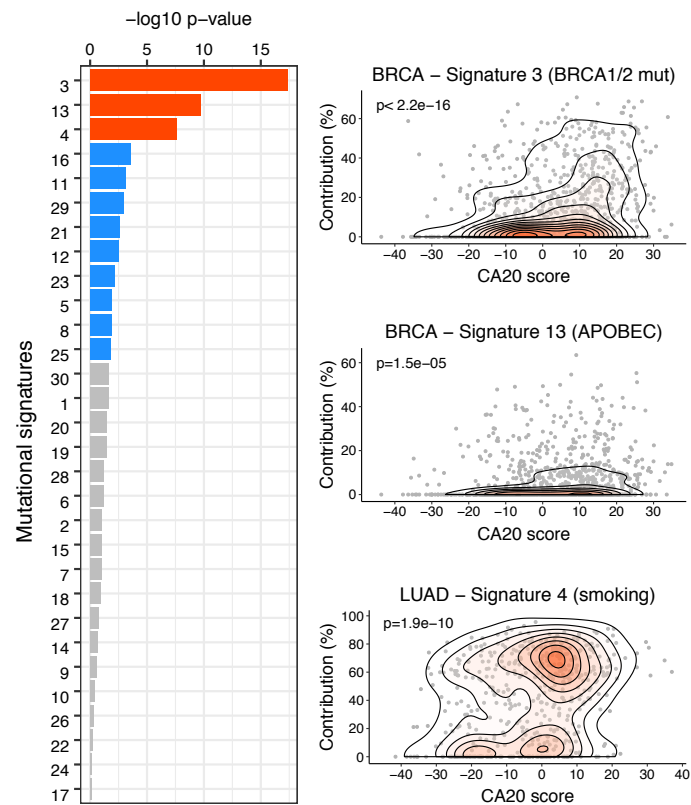

**Supplementary Figure 11: Mutational signatures pan-cancer wide associated with CA20 score.**

Supplement: S11 Fig — Left: Significance of linear regression analyses (-log10 p-value) between CA20 and contribution of each mutational signatures. Positive and negative significant associations (FDR < 0.05) are coloured in red and blue, respectively. Right: Smooth scatter plots showing correlations between CA20 score and contribution of mutational signatures 3, 13 and 4 in breast invasive carcinoma (BRCA) and lung adenocarcinoma (LUAD). Mutational process associated with each signature (in parenthesis) and linear regression p-values are shown. (PDF) [file pcbi.1006832.s011.pdf]

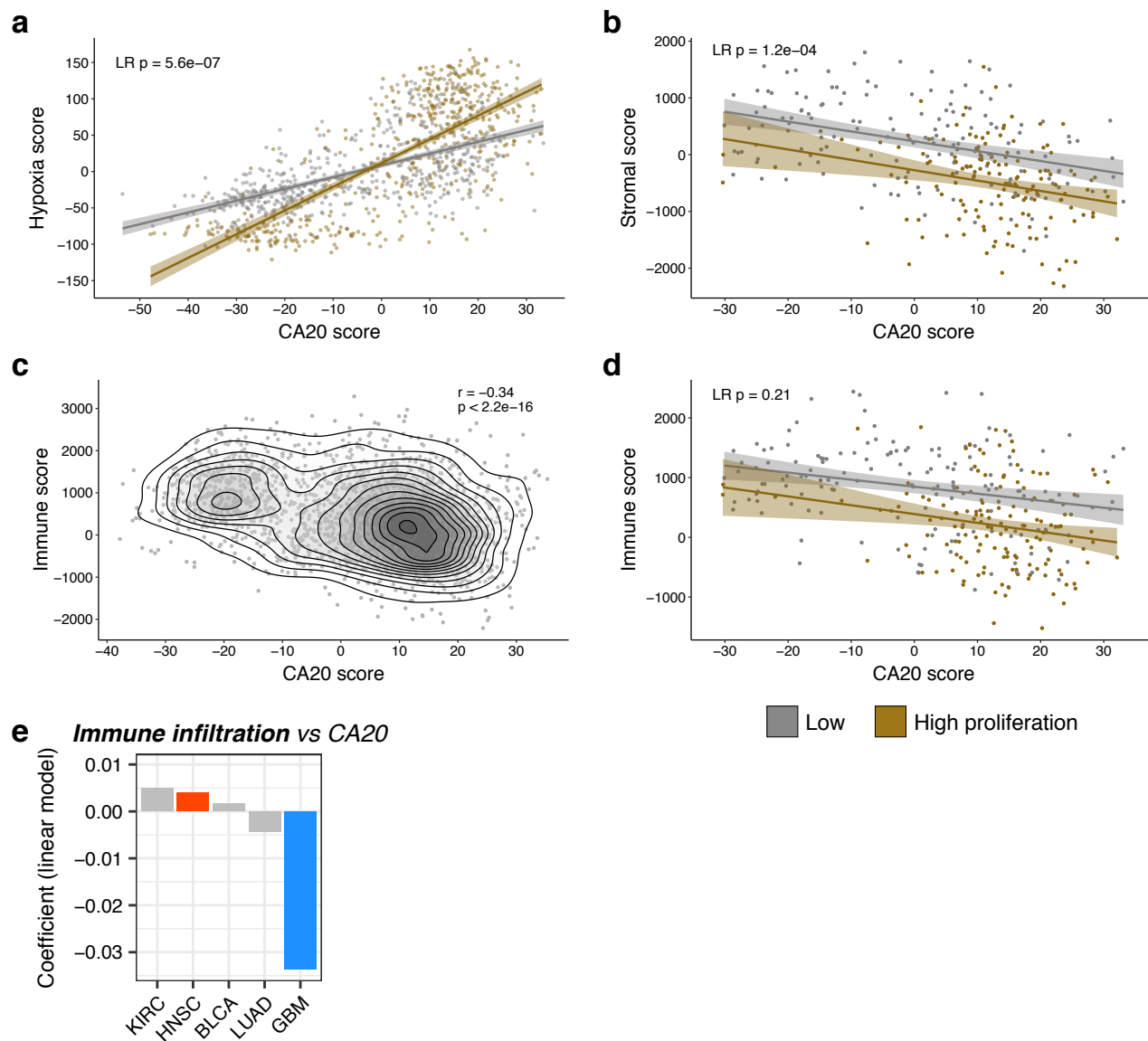

**Supplementary Figure 13:** CA20 is associated with hypoxia and stromal and immune cell infiltration.

Supplement: S13 Fig — (a,b,d) Scatter plots showing correlation between CA20 score and (a) hypoxia, (b) stromal and (d) immune scores across TCGA tumour samples divided in low and high proliferation groups (based on median predicted proliferation rate). Multivariate linear regression (CA20 ~ β0 + β1*hypoxia score + β2*aneuploidy score + β3*mutation burden + β4*CNA + β5*clones per tumour + β6*proliferation rate + β7*cohort or CA20 ~ β0 + β1*stromal score + β2*immune score + β3*aneuploidy score + β4*mutation burden + β5*CNA + β6*clones per tumour + β7*proliferation rate + β8*cohort) p-values for each feature and respective regression lines are shown. Shades around linear regression lines represent their 95% confidence interval. Only samples with information for proliferation rates and genomic instability features were used. (c) Higher CA20 is associated with lower immune cell infiltration. Smooth scatter plot showing correlation between the CA20 and the immune scores across TCGA tumour samples (Spearman’s correlation coefficient, r = -0.34, p-value < 2.2e-16). (e) Higher CA20 is associated with lower immune cell infiltration in glioblastoma and higher infiltration in head and neck squamous cell carcinoma. Linear regression coefficients, representing the CA20 score dependence on the immune score, independently of genomic instability, across the TCGA cohorts with information for all covariates. Significant associations (FDR < 0.05) are coloured. BLCA: bladder urothelial carcinoma; GBM: glioblastoma multiforme; HNSC: head and neck squamous cell carcinoma; KIRC: kidney renal clear cell carcinoma; LUAD: lung adenocarcinoma. (PDF) [file pcbi.1006832.s013.pdf]

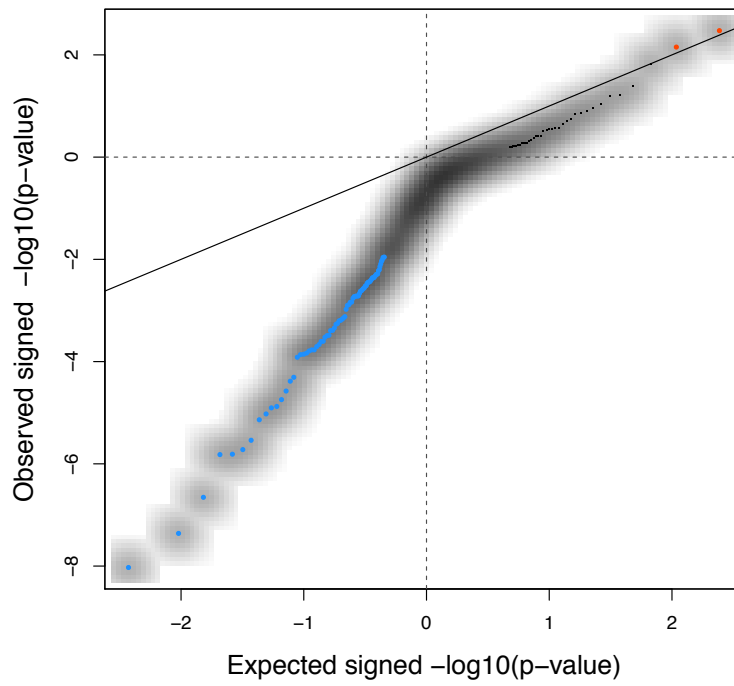

**Supplementary Figure 14:** Q-Q plot of CTRP CA20-AUC Spearman's correlation results.

Supplement: S14 Fig — Quantile-quantile (Q-Q) plot of observed versus expected–log10 of Spearman’s correlation p-values between CA20 and drug-sensitivity (in AUC), with positive or negative sign if the correlation is positive or negative, respectively, across the Cancer Therapeutics Response Portal (CTRP) human cancer cell lines for 354 compounds. The solid line in the Q-Q plot indicates the distribution of compounds under the null hypothesis of no correlation. The compounds whose activity was associated with high and low CA20 (FDR < 0.05; Fig 6a) are represented in blue and red, respectively. (PDF) [file pcbi.1006832.s014.pdf]

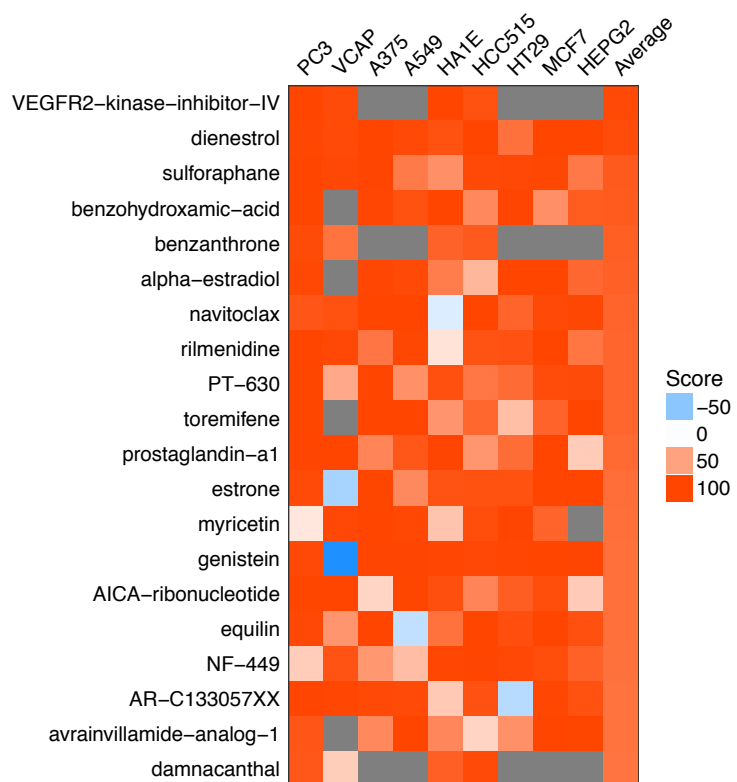

**Supplementary Figure 15:** Compounds that up-regulate the CA20 gene set.

Supplement: S15 Fig — Heatmap of CMap’s drug score, ranging from 100 (maximum CA20 up-regulation) to -100 (maximum CA20 down-regulation) per cell line. Drug average score (last column) is the mean of drug scores across cell lines. The 20 compounds with the highest drug average score are shown and ranked accordingly. Tissue of origin of human cancer cell lines: PC3: prostate; VCAP: prostate; A375: melanoma; A549: lung; HA1E: kidney; HCC515: lung; HT29: colon; MCF7: breast; HEPG2: liver. (PDF) [file pcbi.1006832.s015.pdf]
