# Supplementary material for: Pan-cancer association of a centrosome amplification gene expression signature with genomic alterations and clinical outcome
Source: PLoS Comput Biol. 2019 Mar 11;15(3):e1006832. doi: 10.1371/journal.pcbi.1006832 (PMC6411098; doi:10.1371/journal.pcbi.1006832)
Supplement: S6 Fig — Box plots of CA20 score of TCGA normal samples per alteration (deletion, none, or amplification) of their matched tumour samples on chromosomal arm (a) 5q (n = 297), (b) 16p (n = 566) and (c) 7p (n = 571). All normal samples used here have no CNA in the respective chromosomal arm. * p-value < 0.05 and ** p-value < 0.01 (Wilcoxon rank-sum test). (PDF) [file pcbi.1006832.s006.pdf]

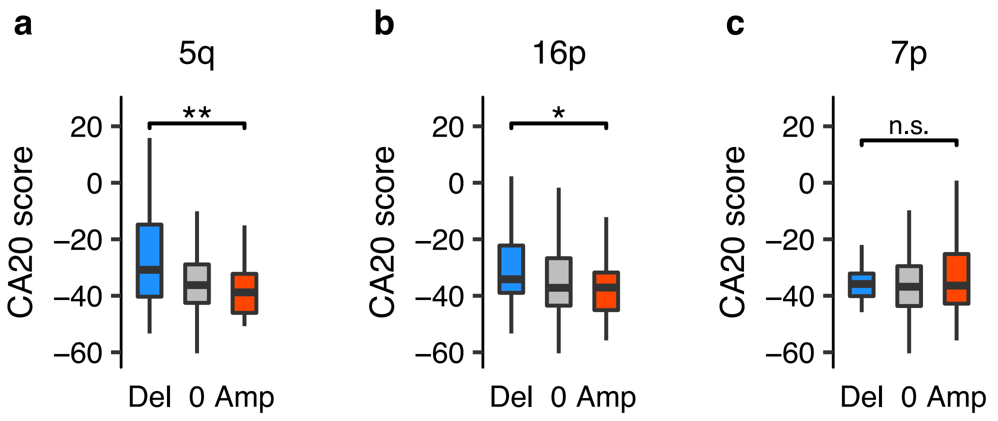

Supplementary Figure 6: Higher CA20 levels in TCGA normal samples whose matched tumours have alterations in 5q and16p chromosomal arms.
