# Supplementary material for: Pan-cancer association of a centrosome amplification gene expression signature with genomic alterations and clinical outcome
Source: PLoS Comput Biol. 2019 Mar 11;15(3):e1006832. doi: 10.1371/journal.pcbi.1006832 (PMC6411098; doi:10.1371/journal.pcbi.1006832)
Supplement: S7 Fig — Scatter plots showing correlation between CA20 score and (a) aneuploidy score (measured as the total number of altered chromosome arms), (b) number of mutations per Mb, (c) number of CNAs and (d) clones per tumour across TCGA tumour samples divided in low and high proliferation groups (based on median predicted proliferation rate). Multivariate linear regression (CA20 ~ β0 + β1*feature + β2*proliferation group + β3*cohort) p-values for each genomic feature and respective regression lines are shown. Shades around linear regression lines represent their 95% confidence interval. Only samples with information for proliferation rates were used. (PDF) [file pcbi.1006832.s007.pdf]

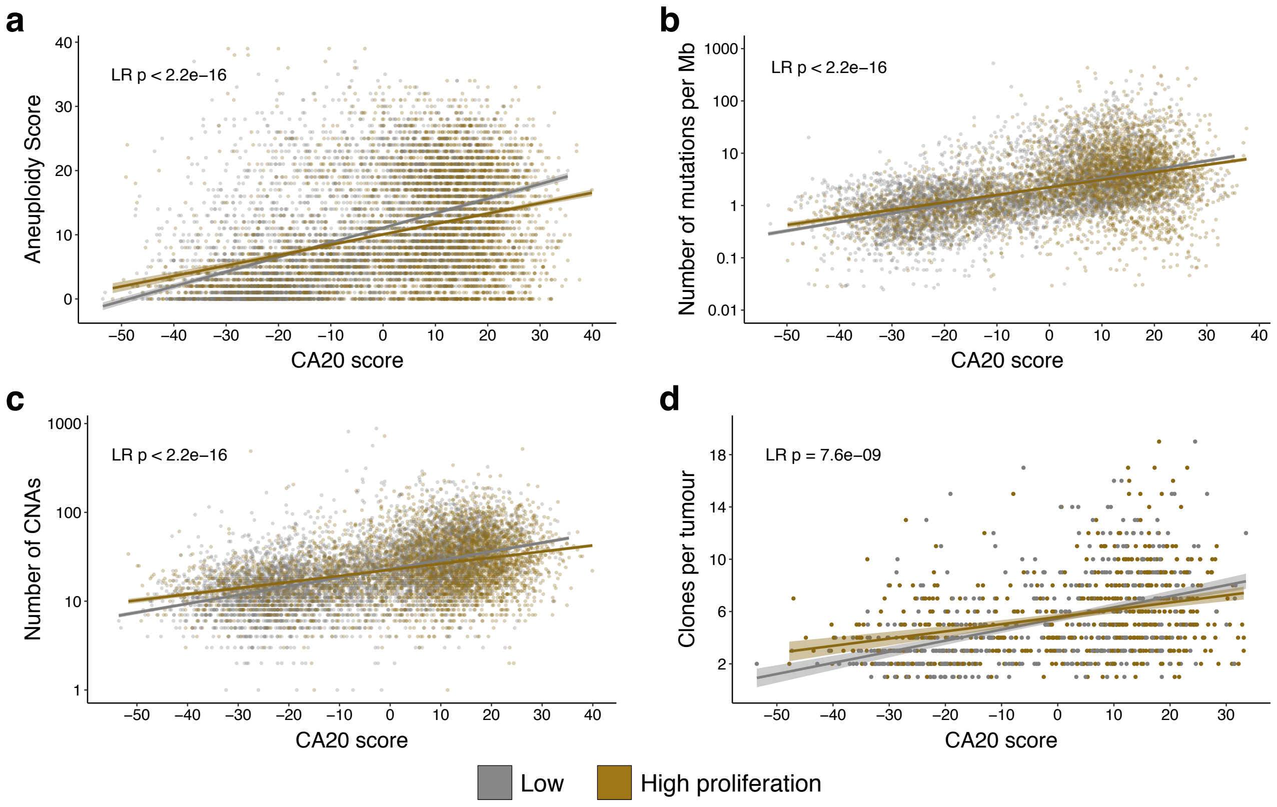

Supplementary Figure 7: CA20 is associated with genomic instability features independently of cell proliferation.
