# Supplementary material for: Pan-cancer association of a centrosome amplification gene expression signature with genomic alterations and clinical outcome
Source: PLoS Comput Biol. 2019 Mar 11;15(3):e1006832. doi: 10.1371/journal.pcbi.1006832 (PMC6411098; doi:10.1371/journal.pcbi.1006832)
Supplement: S10 Fig — (a) Gene Set Enrichment Analysis (GSEA) of genes ranked by their -log10 of linear regression p-value (from Fig 4a) using a list of 299 cancer driver genes derived from [43]. The GSEA p-value is shown. (b) Driver mutations pan-cancer-wide associated with the CA20 score. The volcano plot shows the results of linear regression analyses comparing the CA20 score between wild-type samples and samples with driver mutations for 33 genes (at least 10 samples with driver mutations). Genes whose driver mutations are associated with higher and lower CA20 (FDR < 0.05) are represented in red and blue, respectively. Box plot of CA20 score per TP53 mutation status (wild-type, with passenger, or with driver mutation) is shown. (c) GSEA of genes ranked by their -log10 of linear regression p-value (from Fig 4a) using KEGG pathways. The 15 significantly enriched (FDR < 0.05) pathways are shown and those cancer-associated are highlighted in red. Positively (red) and negatively (blue) enriched pathways, with a FDR lower than 5%, are shown. (d) GSEA plot for the bladder cancer pathway (from c). The GSEA p-value is shown. (e) GSEA of genes ranked by their -log10 of linear regression p-value (from Fig 4a) using MSigDB’s Hallmark Gene Sets. The top 10 gene sets are shown. Only the Wnt/β-catenin signalling gene set is significantly enriched (FDR < 0.05; dark grey). (f) GSEA plot for the Wnt/β-catenin signalling gene set (from e). The GSEA p-value is shown. (PDF) [file pcbi.1006832.s010.pdf]

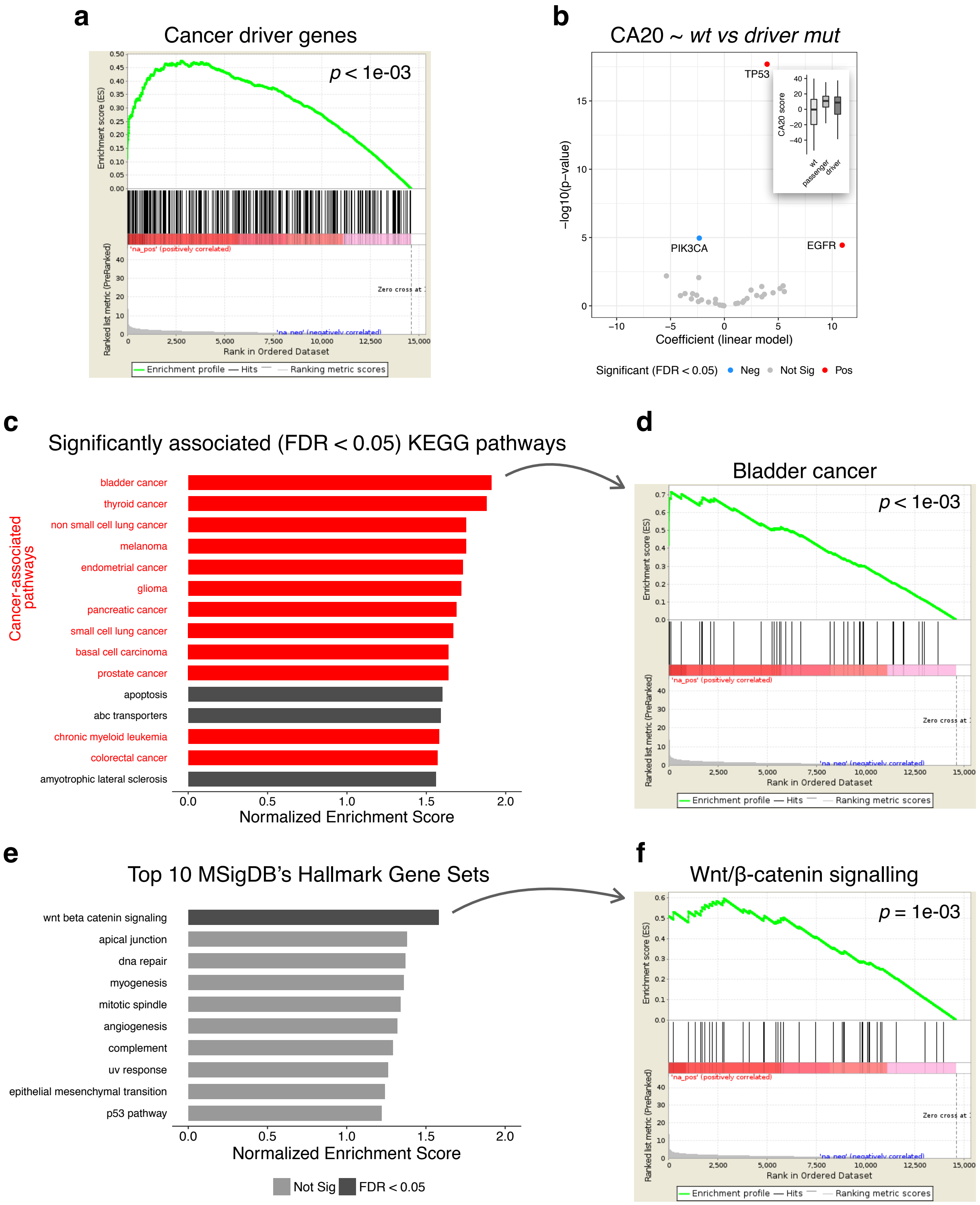

Supplementary Figure 10: Genes whose mutations are associated with CA20 are enriched in cancer driver genes, cancer-associated pathways and Wnt/β-catenin signalling.
