# Supplementary material for: Pan-cancer association of a centrosome amplification gene expression signature with genomic alterations and clinical outcome
Source: PLoS Comput Biol. 2019 Mar 11;15(3):e1006832. doi: 10.1371/journal.pcbi.1006832 (PMC6411098; doi:10.1371/journal.pcbi.1006832)
Supplement: S16 Fig — We used linear regression (gene expression ~ β0 + β1*proliferation rate + β2*cohort) to calculate the association between expression of each compound’s predicted target gene (we merged compound target annotations from the CTRP and CMap datasets) and proliferation rates across TCGA primary tumour samples (S15 Table). (a and b) Scatter plots showing correlations between linear regression coefficient and (a) CMap’s average scores or (b) CTRP’s Spearman correlation coefficients of the respective compounds (Spearman’s correlation coefficient, r = 0.016 and -0.26, p-value = 0.84 and 9e-04, respectively). (c) As in Fig 6d, but with compounds coloured by the linear regression coefficient of the predicted target gene (using the strongest association when a compound has more than one target gene). The two compounds with no annotated target gene are represented in grey. (d) Example for gene RARG. Smooth scatter plot showing correlation between RARG gene expression and predicted proliferation rates of TCGA primary tumour samples. The linear regression p-value is shown. (PDF) [file pcbi.1006832.s016.pdf]

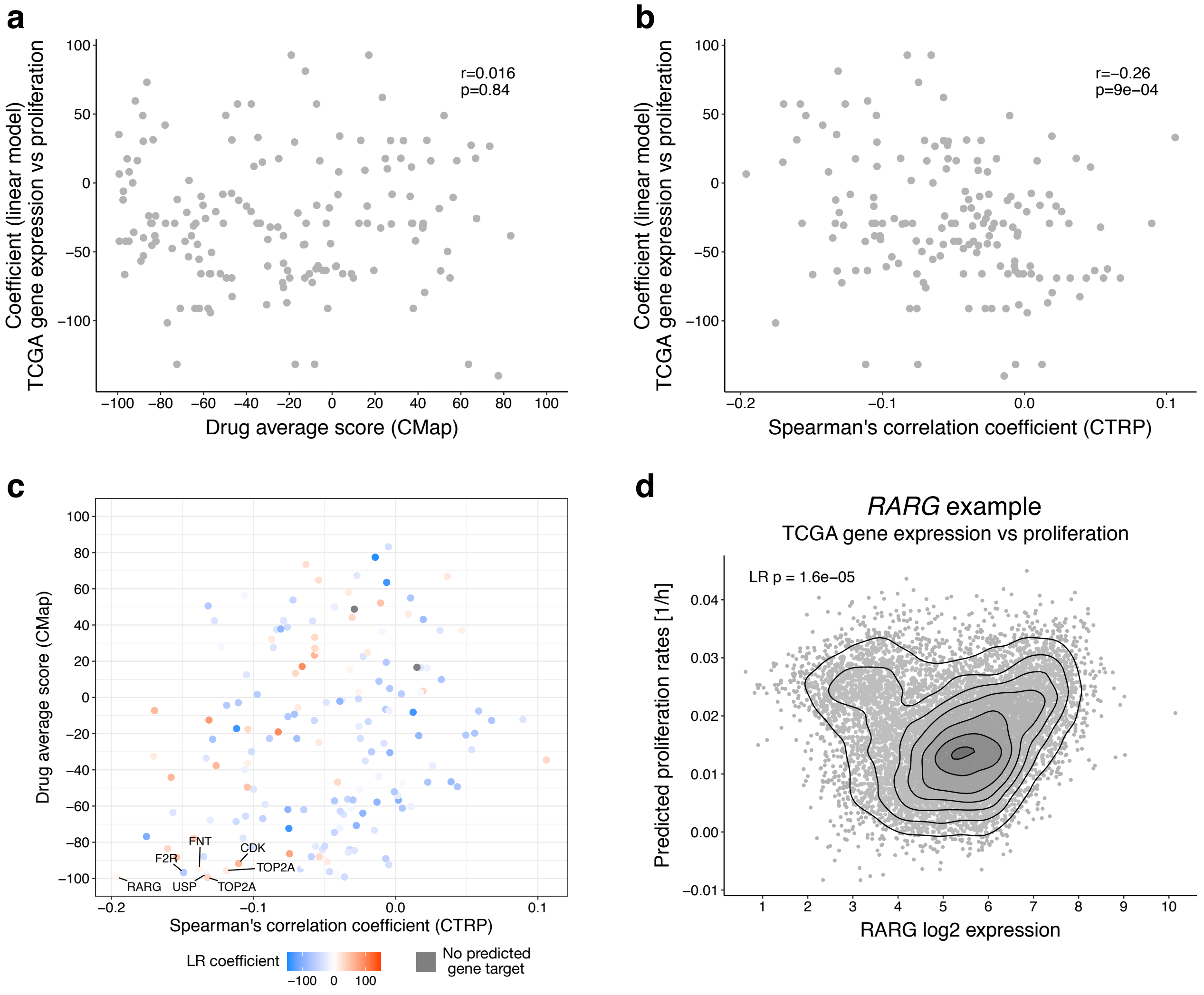

Supplementary Figure 16: Association between compounds' targets and cell proliferation of TCGA samples.
