# Supplementary material for: Pan-cancer association of a centrosome amplification gene expression signature with genomic alterations and clinical outcome
Source: PLoS Comput Biol. 2019 Mar 11;15(3):e1006832. doi: 10.1371/journal.pcbi.1006832 (PMC6411098; doi:10.1371/journal.pcbi.1006832)
Supplement: S17 Fig — (a) CA20 increases from day 0 to day 2, and then decreases to day 4, during multiciliogenesis of adult mouse airway epithelial progenitors cultured in air-liquid interface (ALI). * p-value < 0.05 and ** p-value < 0.01 (Wilcoxon rank-sum test). Data from Mori et al., 2017. (b) Some CA20 genes (PLK4, STIL, CEP152 and SASS6) are significantly upregulated only at the fourth day of multiciliogenesis. Significance of differential expression of CA20 genes between non-ciliating cells and cells undergoing multiciliogenesis harvested at four days (ALI +4), to enrich for genes involved in initial steps of centriole duplication, and twelve days (ALI+12), to enrich for genes expressed when cilia are mature [81]. The Y-axis represents the log10 of FDR-adjusted p-value for differential expression, with positive or negative sign if the sample has higher or lower expression than non-ciliating cells, respectively. Data from Hoh et al., 2012. (c) GSEA of the CA20 gene set on genes ranked by their signed log10 of FDR-adjusted p-value for differential expression, as in (b), between non-ciliating and ALI+4 or ALI+12 cells. GSEA p-values are shown. (PDF) [file pcbi.1006832.s017.pdf]

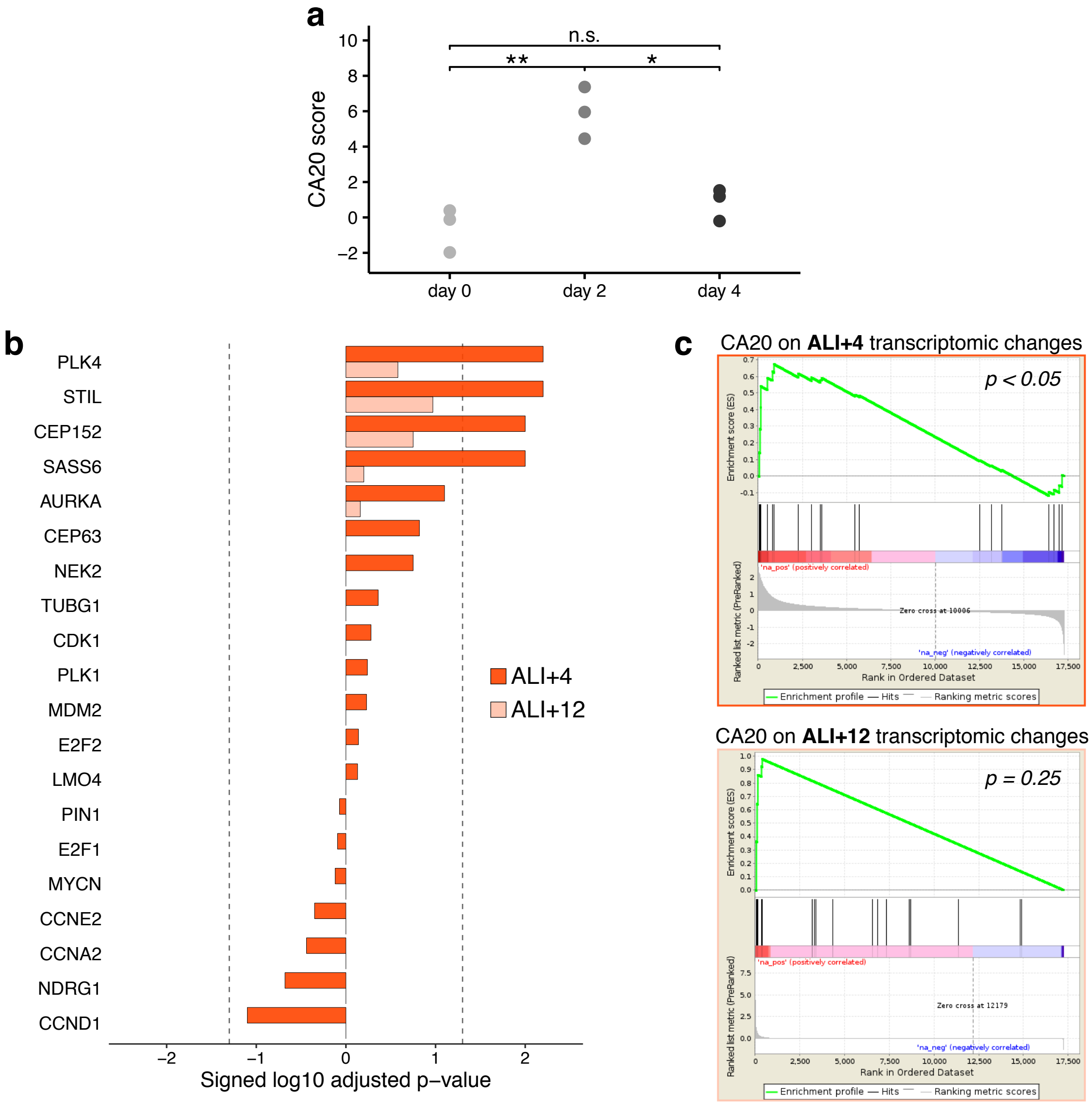

Supplementary Figure 17: CA20 is a surrogate for centriole overduplication during multiciliogenesis.
