# Supplementary material for: Pan-cancer association of a centrosome amplification gene expression signature with genomic alterations and clinical outcome
Source: PLoS Comput Biol. 2019 Mar 11;15(3):e1006832. doi: 10.1371/journal.pcbi.1006832 (PMC6411098; doi:10.1371/journal.pcbi.1006832)
Supplement: S12 Fig — (a and b) Percentage of mutations attributed to each of the 96 substitutions, defined by the substitution class and sequence context immediately 5′ and 3′ to the mutated base, in mutational signatures (a) 1 and (b) 12. The probability bars for the six substitution classes are displayed in different colours. Images retrieved from https://cancer.sanger.ac.uk/cosmic/signatures. (PDF) [file pcbi.1006832.s012.pdf]

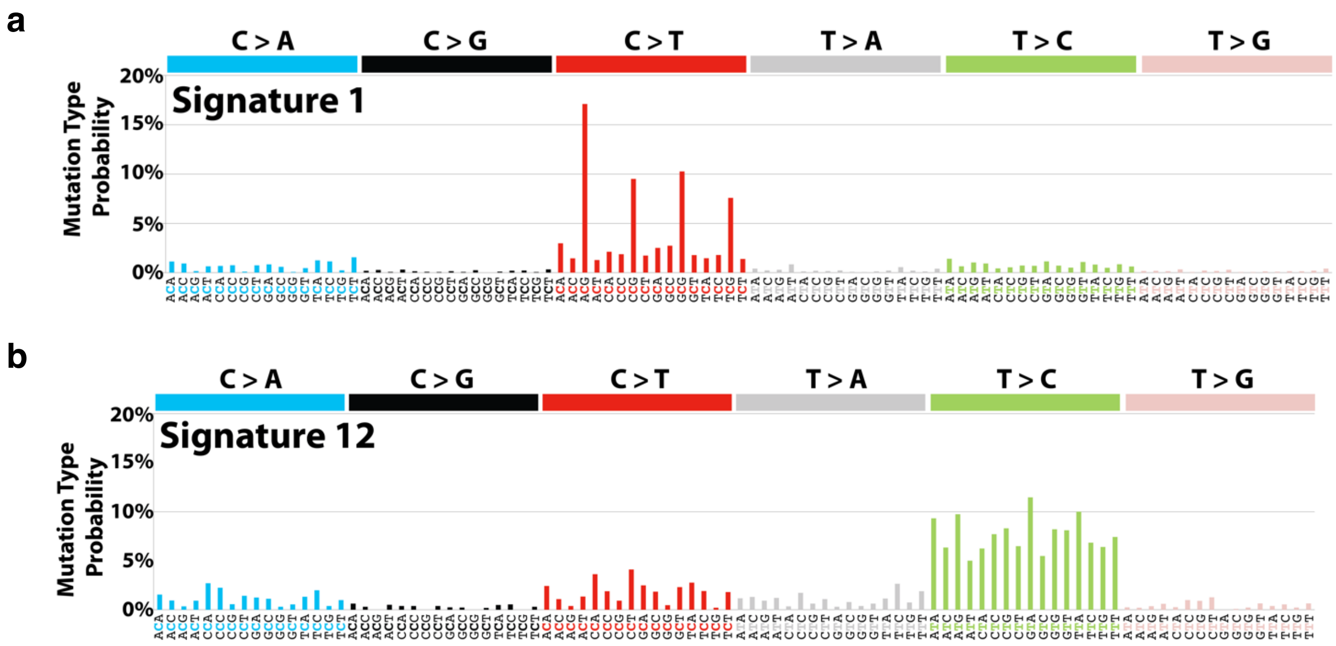

Supplementary Figure 12: Patterns of mutational signatures 1 and 12.
